# Behavioural Characterisation of *Macrod1* and *Macrod2* Knockout Mice

**DOI:** 10.3390/cells10020368

**Published:** 2021-02-10

**Authors:** Kerryanne Crawford, Peter L. Oliver, Thomas Agnew, Benjamin H. M. Hunn, Ivan Ahel

**Affiliations:** 1 Sir William Dunn School of Pathology, University of Oxford, Oxford OX1 3RE, UK; kerryanne.crawford@path.ox.ac.uk (K.C.); thomas.agnew@path.ox.ac.uk (T.A.); 2 Department of Physiology, Anatomy and Genetics, University of Oxford, Parks Road, Oxford OX1 3PT, UK; p.oliver@har.mrc.ac.uk (P.L.O.); benhunn@me.com (B.H.M.H.); 3 MRC Harwell Institute, Harwell Campus, Didcot OX11 0RD, UK

**Keywords:** ADP-ribosylation (ADPr), MARylation hydrolases: Macrod1, Macrod2, behaviour, motor-coordination, gait, hyperactivity

## Abstract

Adenosine diphosphate ribosylation (ADP-ribosylation; ADPr), the addition of ADP-ribose moieties onto proteins and nucleic acids, is a highly conserved modification involved in a wide range of cellular functions, from viral defence, DNA damage response (DDR), metabolism, carcinogenesis and neurobiology. Here we study MACROD1 and MACROD2 (mono-ADP-ribosylhydrolases 1 and 2), two of the least well-understood ADPr-mono-hydrolases. MACROD1 has been reported to be largely localized to the mitochondria, while the *MACROD2* genomic locus has been associated with various neurological conditions such as autism, attention deficit hyperactivity disorder (ADHD) and schizophrenia; yet the potential significance of disrupting these proteins in the context of mammalian behaviour is unknown. Therefore, here we analysed both *Macrod1* and *Macrod2* gene knockout (KO) mouse models in a battery of well-defined, spontaneous behavioural testing paradigms. Loss of *Macrod1* resulted in a female-specific motor-coordination defect, whereas *Macrod2* disruption was associated with hyperactivity that became more pronounced with age, in combination with a bradykinesia-like gait. These data reveal new insights into the importance of ADPr-mono-hydrolases in aspects of behaviour associated with both mitochondrial and neuropsychiatric disorders.

## 1. Introduction

ADP-ribosylation (ADPr) is a modification of proteins and nucleic acids that controls multiple processes common to all kingdoms of life [1,2,3,4]. ADPr is the addition of one (mono) or more (poly) ADP-ribose units using nicotinamide adenine dinucleotide (NAD^+^) as a substrate onto molecular targets, known as MAR- or PARylation and their products as e (mono-ADP-ribose) or PAR (poly-ADP-ribose), respectively. Several families of enzymes can synthesise ADPr, the best understood of which are the poly(ADP-ribose) polymerases (PARPs). PARPs have 17 known family members in humans and are considered the major ADPr contributor in cells; PARP1 accounting for some 85–90% of NAD^+^ consumption [5,6]. ADPr is involved many essential cellular processes such as DNA repair, chromatin remodelling, antiviral responses and WNT signalling [5,7,8,9,10].

Similar to other modifications, ADPr is fully reversible [11]. Removal of ADPr is performed by two main types of ADPr hydrolase families: the macrodomains and ARHs (ADP-ribosylhydrolases) [12]. There many different specificities for hydrolases, dependent upon specific macromolecule modification and the nature of the chemical bond on the macromolecular targets [12,13,14,15,16]. Among the macrodomain family are three enzymes, MACROD1, MACROD2 and TARG1 (terminal ADP-ribose protein glycohydrolase); these share very similar biochemical activities and are responsible for removal of the terminal ADP-ribose moiety from acidic residues [17,18,19]. Interestingly, in terms of neurobiology and behaviour, genetic deficiency of ADPr hydrolases often leads to disease, most commonly neurodegeneration [17,20,21,22,23].

Probably the most poorly understood human ADPr hydrolases are MACROD1 and MACROD2; both share a catalytic macrodomain fold [24] but have different regulatory regions, yet their functions and substrate specificities remain unclear. Both MACROD1 and MACROD2 hydrolyse various mono-ADP-ribosylated substrates in vitro: proteins [18,25,26], nucleic acids [27,28,29] and the O-acetylADP-ribose sirtuin byproduct [30].

Localization of MACROD1 seems largely in mitochondria [29,31] and thus it follows that MACROD1 is highly concentrated in skeletal muscles [29]. Since ADPr- mono-hydrolase activity can be highly specific [32] and perhaps dependent on certain cell types and/or stress, Žaja et al. studied the effect of *MACROD1* loss in a skeletal muscle cell line (RD cells—rhabdomyosarcoma) [33]. Genetic knockout of *Macrod1* in RD cells did not result in any major growth defects but did subtly alter mitochondrial structure [33], which is in-line with our previous work that demonstrated *Macrod1* KO mice are healthy and viable [29]. Previous in vitro studies of MACROD1, using different cell types, have reported MACROD1 as an essential cofactor for androgen receptor [34], estrogen receptor [35,36] and NFκB (nuclear factor kappa-light-chain-enhancer of activated B cells) [37,38] transcriptional activity. Meanwhile, the only confirmed in vivo target of MACROD1 is the aryl hydrocarbon receptor (AHR), working opposite PARP7 (also known as TIPARP), in the toxic response to dioxin [39], which links back again to the mitochondria [40]. Dysfunction of the mitochondria and NAD^+^ metabolism is a common mechanism in neurodegeneration [41,42,43].

On the other hand, MACROD2 resides largely in the cytoplasm [31,33] and is highly expressed in the brain [44]. Indeed, several genome–wide association studies (GWAS) have reported links between *MACROD2* and various neurological and psychiatric conditions such as autism [45], schizophrenia [46], ADHD [47] as well as congenital heart defects [48] (28% of which are linked to neurodegeneration [49]). However, there has been some conflicting reports, for example, *MACROD2* is considered a hotspot for mutation [50] and subsequent studies (using different populations, i.e., Chinese) showed no association with autism [51]. Another role suggested for MACROD2 is in the DNA damage response [52] with ATM (ATM serine/threonine kinase) DNA repair kinase controlling the shuttling of MACROD2 between the cytoplasm and nucleus. Interestingly, deregulation of both MACROD1 and MACROD2 has also potentially been linked to cancer [53,54,55], although the validity of this has been queried [56]. Another suggested biological target of MACROD2 includes glycogen synthase kinase 3β (GSK3β), which is involved in WNT-signalling [25].

Therefore, based on the above information, which suggest an important functional role for MACROD1 in skeletal muscles in cells and a link between *MACROD2* and various neurological conditions, we decided to understand the physiological role of MACROD1 and MACROD2 hydrolases by subjecting knockout mice to a phenotyping battery to assess multiple, spontaneous aspects of mouse behaviour including locomotor activity, anxiety, motor coordination, grip strength, short-term memory and attention.

Genetic KO models for these genes are available on a cost-recovery basis from the International Mouse Phenotyping Consortium (IMPC) and some preliminary phenotyping is already available on their website [57] (www.mousephenotype.org—accessed 7 December 2020). Briefly, the IMPC website shows, *Macrod1* KO was mostly non-significant. While *Macrod2* KO reported phenotypes included a variety of metabolic defects; smaller size, increased circulating blood glucose etc. as well as an abnormal locomotor behaviour (KO mice move further in a light-dark test, on both sides of the light-dark box) and an abnormal sleep pattern [58]. Whilst informative, the information gathered from the IMPC is limited, in particular with regards to later time-points. Age is an extremely important consideration for neurodegenerative disorders, where motor-function and/or mental cognition often gets worse with age [59,60,61]. Specifically for *Macrod1*, mitochondrial decline is age-related [43] hence we chose later time points (12 and 18-months). Whereas *Macrod2* has been associated with ADHD which presents differently in childhood [62], hence why the most practicable youngest age (around three months, at 8–12 weeks) was chosen for the *Macrod2* starting time point, as well as a later time-point (13 months) to follow up.

Our behavioural characterisation revealed interesting and novel phenotypes related to the loss of both *Macrod1* and *Macrod2*. Loss of *Macrod1* resulted in a female-specific motor-coordination defect, whereas *Macrod2* disruption was associated with hyperactivity that became more pronounced with age, in combination with a bradykinesia-like gait. These data reveal new insights into the importance of ADPr-mono-hydrolases in aspects of behaviour associated with both mitochondrial and neuropsychiatric disorders.

## 2. Materials and Methods

### 2.1. Animals and Housing

*Macrod1* and *Macrod2* KO mouse models were obtained via the IMPC [57,63,64]. The *Macrod1* KO strain is a Knockout Mouse Programme (KOMP)-Regeneron (Velocigene) definitive null design (Project ID:VG13617) whereby most of exons 1 through to 3 are deleted and replaced with a promoter-driven Zen_Ub1 cassette [29]. In total 9303 bp were deleted between positions 7131384–7140686 of Chromosome 19 (Genome Build37). This original ‘tm1a’ allele contains a potentially confounding promoter-driven neomycin cassette that was removed by crossing to a *Sox2* promoter-driven cre-recombinase line (*Sox2Cre*, a gift from Elizabeth Robertson, The University of Oxford [65]) to generate the experimental ‘tm1d’ KO allele. The *Macrod2* KO strain is also a Velocigene definitive null design, whereby 19.2 kb (including part of exon 2) is deleted and replaced with a promoter-driven Zen_Ub1 cassette (Project ID:VG12650). In total 19,224 bp were deleted between positions 140226712–140245935 of Chromosome 2 (Genome Build37). Experimental animals were rederived directly from an already-made ‘tm1d’ stock. No overt phenotypes were noticeable from cage-side observations and all mice appeared to age normally.

**Figure 1:**
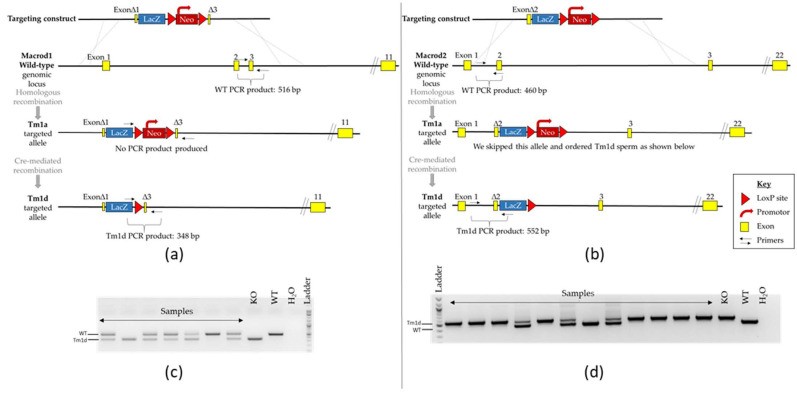
Confirmation of *Macrod1* (left panel) and *Macrod2* (right panel) genetic disruption. (**a,b**) Schematic diagram of the targeting strategy of the WT genomic locus and associated alleles for both genes of interest; note that only mice carrying the tm1d allele were used for behavioural testing. Not to scale. (**c**,**d**) Example of PCR genotyping reactions. DNA fragments were separated on a 2% agarose gel by electrophoresis. Expected product sizes are *Macrod1*: 516 and 348 bp and *Macrod2*: 460 and 552 bp (WT and KO respectively), presence of a double band indicates a heterozygous animal.

The genotype of individual animals was confirmed by PCR on genomic DNA extracted from ear-notches using the Phire Animal Tissue Direct PCR Kit (Thermo Fisher Scientific, Life Technologies Ltd, Paisley, UK) as follows: *Macrod1* primers 5′-AAGCATGGAGGGCATTTTGG, 5′-GGTCCTAAGGTAGCGACTCG and 5′-TGTGGCTTCATTCCAGACAG amplifies products of 516 and 348 bp (wild-type (WT) and KO respectively). *Macrod2* primers, 5′-TTCCTGAGCTCCGTGAATG, 5′-GCAGCAGCTTCCTGAAACAT and 5′-GTCTGTCCTAGCTTCCTCACTG amplifies products of 460 and 552 bp (WT and KO respectively)—see Figure 1 for a scheme.

Mice were weaned at three weeks and kept in same sex groups in controlled conditions (12 h light–dark cycle 09:00–21:00, at 21–22 °C with food and water ad libitum). Cohorts of at least 10 age-matched (within three to four weeks), per sex, per genotype (WT and KO) littermates were analysed in the study from heterozygous (HET) intercrosses. Mice from the IMPC were on a C57BL/6N genetic background, however our in-house C57BL6 mice (including the *Sox2-Cre*) are the C57BL/6J substrain, therefore following at least one backcross our mice were on a mixed C57BL6/J-N genetic background.

All studies were conducted under a valid UK Home Office Animal Project Licence (PPL: 30/3307) which has undergone ethical review by departmental AWERB (Animal Welfare and Ethical Review Body) at the University of Oxford. All work was in accordance with the UK Animals (Scientific Procedures) Act 1986 Amendment Regulations 2012 (ASPA 2012).

### 2.2. Behavioural Testing

The *Macrod1* cohorts were tested at both 12 and 18 months of age (two separate cohorts), while the *Macrod2* cohorts were tested at three and 13 months (one cohort, repeated testing, Table 1). We refer to the cohorts by age at the start of testing throughout in order to distinguish each experimental group/round.

**Figure 2:**
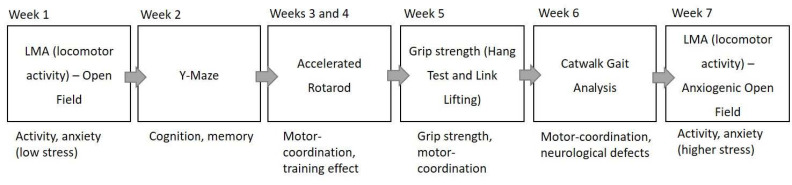
Scheme of behavioural testing overview. NOTE: *Macrod1* KO did not have the second week of accelerated rotarod testing.

All behavioural testing occurred between 12:00 and 17:00 and allowed the animals to acclimatise to the behavioural testing room for at least 30 min. Mice underwent a battery of six tests, one test per week over the course of seven weeks (see Figure 2). Testing was performed by the same scientist to reduce user and handling bias and the experimenter was blinded to genotype. Mice were excluded from testing if they appeared unwell for any reason, for example dermatitis or cataracts in aged mice.

#### 2.2.1. Locomotor Activity (LMA)—Open Field

Mice were placed individually in transparent cages (25.4 cm × 47 cm) containing fresh sawdust. The cage was crisscrossed with 4 × 8 evenly spaced infrared beams. The number of beam breaks in the periphery (outermost beams) and centre (innermost 2 × 6 quadrant) were recorded in 10 s intervals using a beam splitter (San Diego Instruments PAS, San Diego, CA, US) for a total of 90 min. Data examined are total beam breaks and central proportion (%).

#### 2.2.2. Y-Maze—Preference Test

The Y-maze consisted of three clear Perspex arms (30 cm × 8 cm × 20 cm) at 120° to one-another with a central zone [66]. The test was performed in two stages. For the habituation stage the mouse was placed at the end of one (start) arm whilst the entry to one arm is blocked and the second (other) arm is open. Activity was recorded for the initial 5 min after the first entry in to the central zone. The mouse was then returned to its home cage for 1 min. Stage two: the mouse was returned to the maze at the end of the same start arm with the third (novel) arm now accessible. Activity was recorded for 2 min after entry into the central zone. Start arm and closed arm were rotated, in a logical ordered fashion, between each mouse (since experimenter was blinded to genotype and mice were kept in cages with mixed genotypes) to reduce room positional bias. Activity data were quantified from an overhead camera using ANY-Maze software (Stoelting, Dublin, IE). The distance travelled, the time spent in each of the arms, the number of arm entries and the number of repeat entries to the same arm were recorded. The preference ratio was calculated as the time in the novel arm/(time in novel arm + other arm).

#### 2.2.3. Accelerating Rotarod

A commercial rotarod device was used (Med Associates, Inc., Fairfax, VT, USA) consisting of a grooved plastic beam 5 cm in diameter (with dividers to stop physical interactions between animals). Mice were placed on the beam (revolving at the default 5 rpm) facing in the opposite orientation to rotation. After 30–60 s to allow mice to become accustomed to walking on the beam (and to load multiple mice, up to five at a time) the speed was gradually accelerated to a maximum of 50 rpm over 5 min by electronic control of the motor. The latency before falling was measured up to a maximum total time of 5 min. Trails were repeated three times in total over three consecutive days. For *Macrod1*, the average time taken to fall from the three days is presented. For *Macrod2* cohort only, because many of the female mice could stay on for the full 5 min, a final test was performed the following week at a faster speed 8–80 rpm, to avoid a ceiling affect with analysis. The *Macrod2* final trial was performed on a single day, three runs with one hour’s rest between trails, the mean of which is presented. Data examined are average latency to fall (s).

#### 2.2.4. Grip Strength

##### Inverted Screen—Four-Limb Hang Test

Mice were placed on the centre of a metal grid (12 mm^2^ of 1 mm diameter wire, 45 cm^2^ in size within a 5 cm wooden frame). The grid was then inverted over a padded surface. The time taken to fall from the grid was recorded over three trials in total over three consecutive days. The data are presented as the square root transformed hanging impulse (gs) = hang time (s) × mass (g). There was no maximum time set for *Macrod1* (most mice fell off within 2–3 min, with only one or two mice lasting longer than 5 min). However, no maximum time was considered impractical for the three-month-old *Macrod2* cohort, where many mice could hang 10+ min. Therefore, we decided instead to screen *Macrod2* mice for only 5 min as a benchmark (nearly all mice passed) and then use a different method to assess grip strength (see Link Lifting below) to avoid a ceiling effect in analysis.

##### Link Lifting

Mice were allowed to grip onto wire balls for kettle descaling that were attached to a varying number of metal chain links of increasing weight [67]. Starting with the lowest weight, mice were allowed to grip the wire ball their front paws only, a stop watch was started and then they were lifted gently by the tail. Mice had three chances to lift the links just clear of the bench, for a criterion time of 3 s (as determined by stopwatch) before trying the next heaviest weight. For mice that failed to lift one set of links, the longest attempt was recorded and used as a score qualifier. A lift score was calculated as: (max link lifted × 3) + (next link × time held in seconds).

#### 2.2.5. Catwalk Gait Analysis

Mice were placed on a narrow, sealed, raised platform (10 cm × ~1 m), and their activity is recorded using a camera placed below the floor (Catwalk XT, Noldus). The Catwalk system uses illuminated footprint technology, in which, briefly, the walking surface is made of glass which internally reflects a green LED light, except where a paw touches the glass, thus footprints or other miscellaneous points of contact glow green. The test is performed in the dark; five compliant runs of ~55 cm in length (six to seven step cycles) were taken from each mouse. Compliant runs were determined as maximum duration 5 s and maximum speed variation of 35%. Compliant runs are classified, and the following data was examined; maximum contact mean intensity (forepaw and hind paw, average of left and right sides used square root transformed), gait velocity (cm/s), gait cadence (steps/minute–, square root transformed), forefoot swing speed (cm/s), forefoot stride length (cm).

#### 2.2.6. LMA Anxiogenic Open Field—Bright Field

Mice were placed in a white circular drum (60 cm × 60 cm lit by four high-fluorescent 7 W OSRAM Deluxe light bulbs) and their activity recorded for 5 min using a camera placed over the centre of the drum. Automated video tracking software (ANY-Maze, Stoelting, Dublin, IE) was used to record the total distance travelled (m), number of entries into the central zone (20 cm diameter) and time (s) in the central zone (s).

### 2.3. Statistics

The statistical software Minitab 2019 was used to analyse the results. The data were checked for normal distribution by using histogram plots and some data were square root transformed to remove any skewing of data (mentioned in Results, below). Analysis for *Macrod1* and *Macrod2* was performed separately.

Initial analyses included an all-factors-combined analysis of variance (ANOVA) (described in detail below) to give a highly powered statistical overview. If genotype was statistically significant (*p*-value of <0.05) we then performed further post hoc analysis to further clarify that genes’ role. Further tests typically included analysis of covariance (ANCOVAs) to calculate significance, as weight tended to highly influence results [68,69,70,71] and Cohen’s d statistic (d-score) to objectively measure effect size (difference in means ÷ population standard deviation).

Since *Macrod1* was two independent cohorts, we used the general linear model ANOVA (a factorial-ANCOVA which allows use of covariables such as weight), with genotype, sex and cohort (age) as cofactors and weight as a covariable. The following interactions were also included in the model: genotype*sex, genotype*cohort, weight*sex, weight*sex, weight*genotype, sex*cohort. We continued to model the weight*genotype interaction for *Macrod1* because whilst not statistically significant the *p*-value was borderline (see Results below, Table 2).

As the *Macrod2* cohort involved repeated testing of the same mice, we used a repeated measures ANOVA, thus Mouse Name (random factor, nested with genotype and sex) was also analysed along with the above cofactors and interactions listed for *Macrod1* (excluding weight*genotype which was ruled out after an initial weight analysis—see Table 2). Minitab 2019 automatically corrects for missing values.

Results are presented as the mean ± standard error of the mean (SEM). A *p*-value of <0.05 was considered significant. Cohen’s d-score was assigned as follows; <0.2 trivial, >0.2 small, 0.5–0.8 medium and >0.8 large effect size. All graphs were generated using Prism 8 and all significant differences are marked with a solid line and an asterisk. A dashed line was used to mark trends (not significant and no asterisk added). The exact *p*-value can be found in the figure legends as well as various tables.

## 3. Results

### 3.1. Confirmation of Genetic Knock-Out

All *Macrod1* and *Macrod2* KO mice were born at the expected Mendelian ratios. Protein deletion in these genetic models has been reported previously by us for Macrod1 [29] and by two other independent research groups for Macrod2 [53,72].

### 3.2. Weight Evaluation

**Figure 3:**
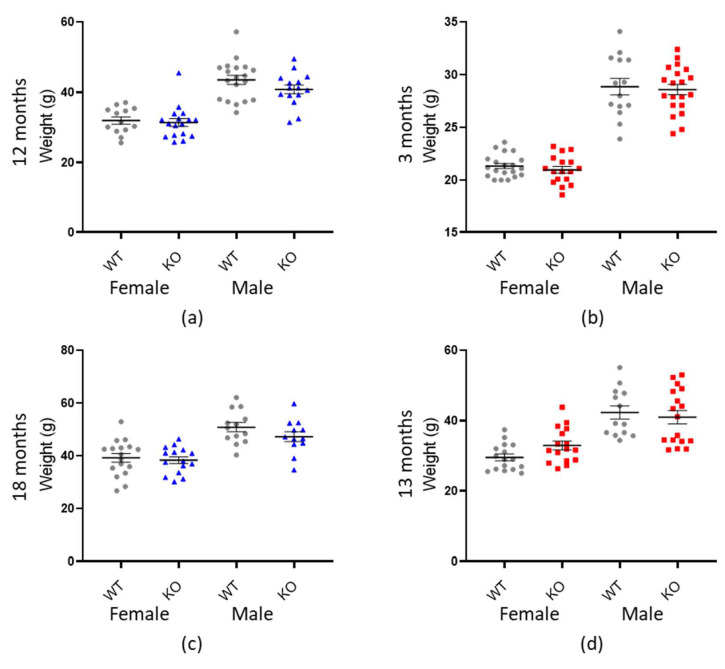
There is no association between weight and genotype. Graphs show individual values dot plot for female and male mice, WT shown in grey circles, *Macrod1* KO in blue triangles and *Macrod2* KO in red squares. Error bars are the SEM. Graphs are as follows: (**a**,**c**) *Macrod1* 12 months and 18 months, (**b**,**d**) *Macrod2* three months and 13 months.

Since many aspects of behaviour are affected by an animal’s weight (such as ambulation, dexterity and strength [68,69,70,71]) it was important to first determine if genotype was significantly associated with differences in mass. In both genotypic cohorts, there was no impact of genotype on weight (*Macrod1*: *p* = 0.058 and *Macrod2*: *p* = 0.701 (Table 2 and Figure 3). Animal weight was, however, significantly affected by sex and age in both groups (*p* < 0.001) as expected [73,74].

### 3.3. Macrod1 KO Is Associated with Sex-Specific Reduced Motor-Coordination

**Figure 4:**
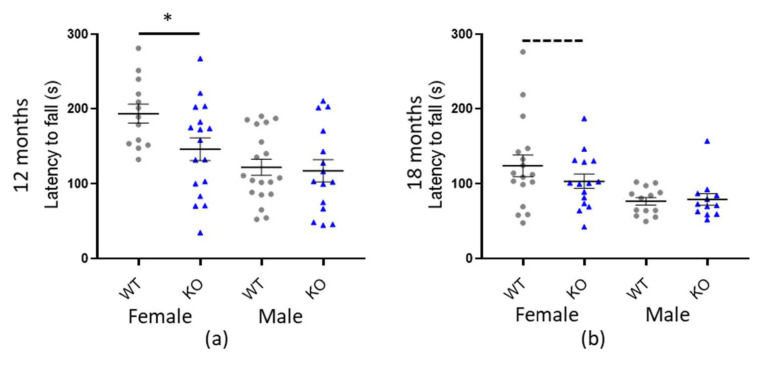
*Macrod1* KO is associated with sex specific reduced fall latency on the rotarod. Graphs show individual values dot plot, separated by sex and cohort, WT shown in grey circles and *Macrod1* KO in blue triangles. Error bars are the SEM. Graphs are as follows, (**a**,**b**) *Macrod1* aged 12 and 18 months. Significant difference for *Macrod1* KO 12 months; F_(1,27)_ = 14.53, * *p* = 0.005 and trending at 18 months; F_(1,29)_ = 3.11, *p* = 0.089 (ANCOVA).

We performed a standard test of mouse motor-coordination, the accelerated rotarod, which records one linear variable, the latency to fall (s). Our initial statistical analysis revealed a significant effect of genotype for *Macrod1* KO (* *p* = 0.018) but not *Macrod2* KO (*p* = 0.357) (see Table 3 and Supplementary Table S1 and Figure S1). Main effect in both cohorts was weight (*p* < 0.001) however the *Macrod1* cohort also had a significant effect with sex (* *p* = 0.006), weight*sex interaction (* *p* = 0.005) and the weight*genotype interaction (* *p* = 0.045). Therefore, we postulated there might be a sex-specific difference. Indeed, further investigation and plotting of the data revealed that the effect was most pronounced in 12-month-old female mice, who fell off the rotarod 47.3 s (±15.2 SEM) sooner (* *p* = 0.005) than their female WT littermates (see Figure 4). Eighteen-month-old female mice followed the same trend and fell 20.62 s (± 9.52 SEM) sooner than their female WT littermates however this was not significant on its own (*p* = 0.089). A 47.3 s reduction in latency to fall for the 12-month old females is considered a medium to large effect size (*d* = 0.788), indicating that this is a relevant biological difference. There were no significant differences in latency to fall between the KO and WT males. Taken together our data demonstrate a moderate female-specific motor-coordination defect in the *Macrod1* KO mice.

### 3.4. Macrod2 KO Increases Total Locomotion Activity

We observed *Macrod1* and *Macrod2* KO mice in a range of spontaneous behavioural testing paradigms. In nearly all tests where total distance travelled was recorded, the *Macrod2* KO mice moved further than their WT littermates, indicating a hyperactivity phenotype. These tests included locomotor activity (LMA), open field (see Table 4), Y-maze preference test (see Table 5) and LMA anxiogenic open field (bright field) (not significant but follows trend—see Supplementary Table S4 and Figure S4).

**Figure 5:**
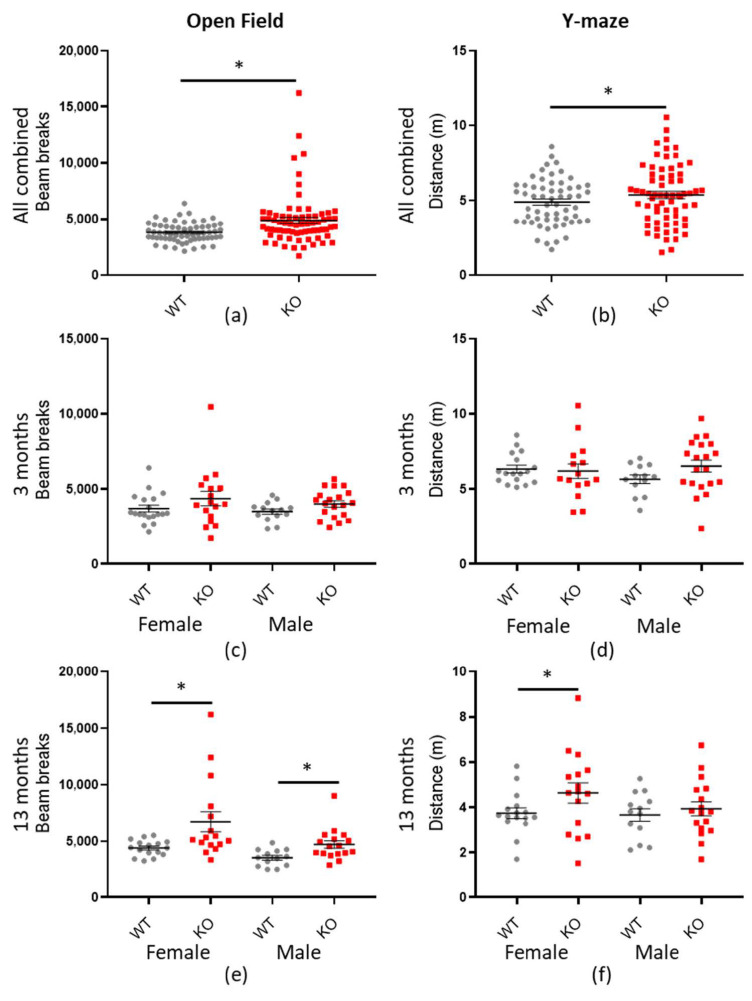
*Macrod2* KO is associated with hyperactivity. Graphs show individual values dot plot for all mice combined or separated by sex and age, WT shown in grey circles and *Macrod2* KO in red squares. Error bars are the SEM. Graphs are as follows: (left panel) LMA open field total beam breaks over 90 min; (**a**) all data combined, (**c**) three months and (**d**) 13 months. (Right panel) Y-maze preference test total distance travelled (m) in 2 min (**b**) all data combined, (**d**) three months and (**f**) 13 months. Significant difference for *Macrod2* KO LMA open field: (**a**) F_(1,55)_ = 12.14, * *p* < 0.001, (**e**) 13-month-old females F_(1,29)_ = 9.24, * *p* = 0.005, males F_(1,27)_ = 6.68, * *p* = 0.015. Significant differences for *Macrod2* Y-maze: (**b**) F_(1,51)_ = 5.74, * *p* = 0.019, (**f**) 13-month-old females F_(1,29)_ = 6.09, * *p* = 0.020.

Our main test of locomotor activity is the LMA open field, which records mice for 90 min. Our initial statistical analysis revealed a highly significant effect on total beam breaks with genotype for *Macrod2* KO (* *p* = 0.001), but not *Macrod1* KO (*p* = 0.427) (see Supplementary Table S3 and Figure S3). Additional significant effects were for genotype*cohort (* *p* = 0.021) and random variable Mouse Name (* *p* = 0.002). Mouse Name is a term included in the *Macrod2* statistical modelling which accounts for repeated testing of individual mice at both time points (see Methods). A significant *p*-value for Mouse Name indicates that the nature of individual mice is consistent between tests (a mouse that moved further at one time-point was highly likely to move further at the other time-point). An effect for genotype*cohort demonstrates an effect of genotype with age. Indeed, a hyperactivity phenotype was actually more pronounced in the aged *Macrod2* KO mice for the LMA open field test (see Figure 5 and Table 4 for a statistical summary). Consistent with this, the three-month-old female and male mice were not significant when examined individually and had a small to medium effect size (d = 0.431 and d = 0.585 respectively). Whereas the 13-month-old *Macrod2* KO female (* *p* = 0.005) and male (* *p* = 0.015) mice were independently significant and had large effect sizes (*d* = 0.840 and *d* = 0.915 respectively). Practically, this translates as the 13-month-old *Macrod2* KO mice moved 33–53% more than their WT counterparts in the LMA open field test.

Similarly, the Y-maze preference test (see Supplementary Tables S6 and S7 and Figures S6 and S7 for preference ratio data—no significant differences) also showed a significant difference in locomotor activity between the genotypes (* *p* = 0.019—see Table 5 for statistical summary). The effect was most pronounced in the 13-month-old female mice (* *p* = 0.020) with a medium effect size (*d* = 0.560), although, the hyperactivity trend was present in all but the three-month-old female mice (see Figure 5). On the other hand, the bright field test did not show any significant differences in total distance travelled (*p* = 0.072), however, the overall trend is still present (see Supplementary Table S4 and Figure S4). Taken together, our results demonstrate that *Macrod2* KO is associated with increased total locomotion activity and that this hyperactivity is more pronounced with age.

### 3.5. Macrod2 KO Is Associated with Reduced Speed and a Shorter Stride Length

**Figure 6:**
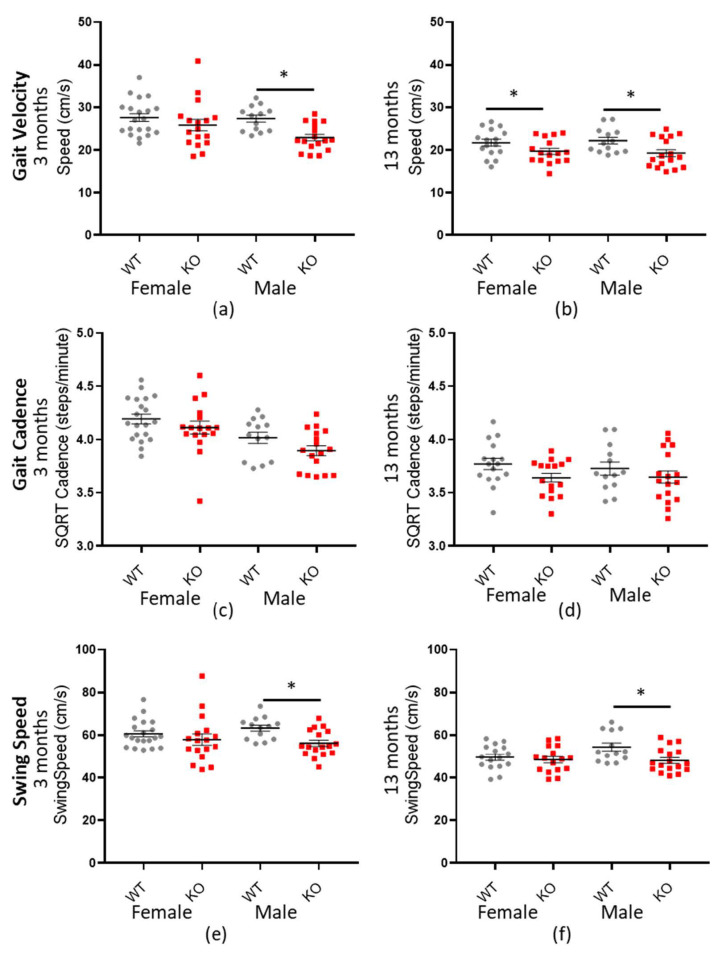
*Macrod2* KO is associated with a slower gait. Graphs show individual values dot plot, separated by sex and age, WT shown in grey circles and *Macrod2* KO in red squares. Error bars are the SEM. Graphs are as follows (**a**,**b**) gait velocity (average speed) at three and 13 months, (**c**,**d**) SQRT gait cadence (steps/minute) at three and 13 months and (**e**,**f**) forepaw swing speed (cm/s) at three and 13 months. Significant differences for *Macrod2* KO (**a**) three-month-old males F_(1,27)_ = 15.09, * *p* = 0.001, (**b**) 13-month old females F_(1,29)_ = 4.33, * *p* = 0.046, males F_(1,27)_ = 7.98, * *p* = 0.009, (**e**) three-month-old males F_(1,27)_ = 10.82, * *p* = 0.003 and (**f**) 13-month-old males F_(1,27)_ = 7.26, * *p* = 0.012.

Mouse ambulatory speed was measured by a number of factors, including gait velocity, gait cadence and forepaw swing speed (see Table 6, Table 7 and Table 8 for a statistical summary). In each case, *Macrod2* KO mice were consistently and significantly slower than their WT littermates overall; gait velocity −2.922 cm/s (± 0.571 SEM) * *p* < 0.001: SQRT-gait cadence −0.952 steps/min (±0.272 SEM) * *p* = 0.026: forepaw swing speed −4.32 cm/s (±1.06 SEM) * *p* = 0.001. When further analyses were conducted, separated by sex and age, the three- and 13-month-old males and the 13-month-old females had the largest effect size. For gait velocity and forefoot swing speed, three-month-old males had the largest effect size (*d* = 1.188 and *d* = 1.053) followed by the 13-month-old males (*d* = 0.842 and *d* = 0.912) whereas the three-month-old females had only small differences. Interestingly, the 13-month-old females had a significant, medium effect size for gait velocity but only a negligible and non-significant difference for forefoot swing speed (see Table 6 and Table 8). Another discrepancy was for gait cadence, which showed a small but significant effect size overall (*d* = 0.420) but not for testing the sexes and ages separately (see Table 7). Nonetheless, the overall trend, for *Macrod2* KO mice to be slower, remained present as can be observed in Figure 6.

**Figure 7:**
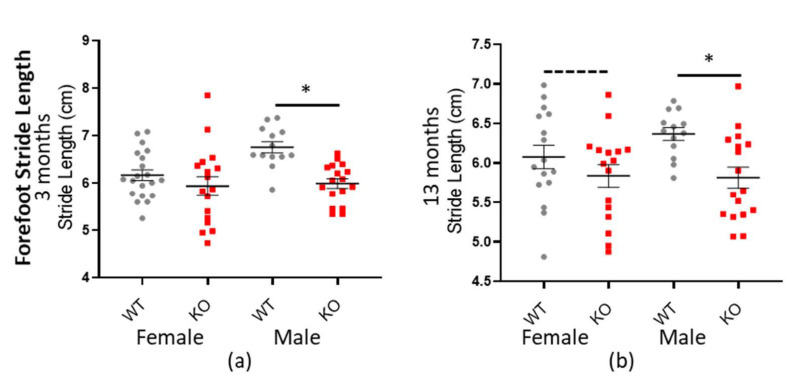
*Macrod2* KO is associated with a shorter stride length. Graphs show individual values’ dot plot, separated by sex and age, WT shown in grey circles and *Macrod2* KO in red squares. Error bars are the SEM. Graphs are as follows: (**a**,**b**) forefoot stride length (cm), three and 13 months. Significant differences for *Macrod2* KO (**a**) three-month-old males F_(1,27)_ = 23.85, * *p* > 0.001, (**b**) 13-month-old males F_(1,27)_ = 7.98, * *p* = 0.009 and trending for 13-month-old females F_(1,29)_ = 3.63, *p* = 0.067.

Additionally, *Macrod2* KO mice have a shorter stride length overall: −0.414 cm (± 0.0734 SEM) * *p* < 0.001 (all factors and ages combined—see Table 9). The genotype*sex interaction was also significant (* *p* = 0.033), implying perhaps a different effect in females vs. males, based on genotype, however when we plotted the data (see Figure 7) it appeared as if *Macrod2* KO display reduced stride length in both the genders, albeit more for the males. Further analysis showed that the female mice had only a small effect size (*d* = 0.345 and *d* = 0.406 for three and 13 months respectively), whilst the male mice had a much larger effect size (*d* = 1.361 and *d* = 1.045 for three and 13 months respectively; see Table 9). The male forefoot stride length data was also independently highly statistically significant (* *p* > 0.001 and * *p* = 0.003 for three and 13 months, respectively). Reduced stride length does not entirely explain the *Macrod2* KO mice decreased average speed, because the gait cadence was also marginally reduced. Only the forepaw data is presented here, however hind paw data followed the same trend. As an additional control, there was no significant differences for these same traits when compared for the *Macrod1* cohorts (see Supplementary Tables S10 and S11 and Figure S9).

In conclusion, *Macrod2* KO mice have an abnormal gait. Peculiarly, since *Macrod2* KO mice were determined to be hyperactive (total distance travelled in a variety of testing paradigms) their natural walk, as recorded by the catwalk test (in the dark, least stressful), was actually slower and with shorter steps. This type of gait is known as bradykinesia (a slow shuffling gait as appears in Parkinson’s disease [75]). Males are possibly more affected than the females, however the trend is present in both sexes.

### 3.6. Further Behavioural Testing Summaries

Key results have been highlighted above however there are some other minor but important points to make as a result of this preliminary behavioural phenotyping. Neither *Macrod1* or *Macrod2* KO appear to have an effect on short-term working memory or attention (no significance difference in the Y-maze preference test—see Supplementary Tables S6 and S7 and Figures S6 and S7) or grip strength (see Supplementary Tables S13 and S14 and Figures S11 and S12) or anxiety levels (LMA open field and bright field—see Supplementary Tables S2–S5 and Figures S2–S5). Additionally, we initially thought there might be an altered gait in *Macrod1* KO mice, since they appeared to place less pressure on their front paws (but not hind paws) when walking (catwalk gait analysis), however further examination determined this phenotype to be due instead to subtle differences in animal weight (see Supplementary Tables S9 and S12 and Figures S8 and S10). Another phenotype we ruled out for *Macrod1* KO was hypoactivity for the 12-month old female mice in the Y-maze test only (see Supplementary Table S8). Whilst the effect size seemed moderate (d = 0.740), the *p*-value was borderline (* *p* = 0.047) and no other hypoactivity traits could be seen in any of the other testing paradigms.

## 4. Discussion

Our study shows the first behavioural characterization of the mono-ADP-ribosylation hydrolyses Macrod1 and Macrod2. Since there is nothing known about the physiological targets of these genes and muscular or neurological functional roles are suggested, we decided to investigate them further with some standard spontaneous behavioural testing in KO mouse models.

Genetic loss of *Macrod1* resulted in a female-specific motor-coordination defect. The mild to moderate motor-coordination defect observed, whereby *Macrod1* KO female mice fell 20–47 s sooner from a rotating rod than their WT littermates, was not likely due to a fundamental difference in grip strength as no differences were measured in the inverted hang test. Additionally, this reduced latency to fall was not obviously due to an abnormal gait, as no differences between genotypes were observed across several parameters during the catwalk test.

Previous studies have demonstrated that reduced latency to fall from the rotarod can be a result of muscle disease [76], mitochondrial disease [77] as well as mitochondrial dysfunction induced by ischemic injury [78]. Therefore, one plausible explanation for *Macrod1* KO’s reduced latency to fall from the rotarod, based on the mitochondrial subcellular location of Macrod1 and enrichment in skeletal muscles [29,33] could be, loss of aerobic (mitochondrial) fitness. Indeed, Loss of *MACROD1* in RD cells has been shown to cause mitochondrial fragmentation [33] which might become a problem under continued muscle use and increasing energy demands [79], such as the rotarod test. Certainly, altered mitochondrial structure has previously been linked to muscle fatigue [80]. Since *Macrod1* KO mice are viable, fertile and healthy it is logical that perhaps MACROD1 is only activated under certain conditions, such as stress or exercise. Further supporting a role in exercise, Macrod1 appeared specifically upregulated as part of evening (but not morning) exercise adaptation in rats [81].

Following on, the intriguing sex-specific differences we see, as well as the smaller difference in the 18-month old females could simply be a matter of shorter exercise duration and thus less mitochondrial energy demands [79]. Males (both ages) and the aged females spend approximately half the total time on the rotarod compared to the 12-month-old WT female mice, it is possible that the phenotype only presents following longer periods of exercise, as in the 12-month-old WT female mice, upon which aerobic mitochondrial respiration takes over from the initial reliance on glycolytic respiration.

Alternatively, sex-specific alterations in *Macrod1* regulation in vivo are potentially consistent with *Macrod1* being an estrogen and androgen sensitive gene [34,35,36]. There are broad influences of sex hormones within the brain, which can influence many aspects of behaviour [82]. Interestingly, with regards to the mitochondria, non-nuclear estrogen receptors have been documented in close proximity to this organelle [83]. Additionally, there are physiological muscular differences between male and female mice, for example higher mitochondrial content in female mice which likely contributes to their increased rotarod ability and, therefore, lack of said mitochondrial benefit may underpin the reduced rotarod latency to fall observed in *Macrod1* deficient females [84].

Could our observed phenotype be due to a subtle maladaptation in mitochondria which only shows up under physical exertion or perhaps other stresses? We did not perform any of the more aversive ‘exhaustion’ type behavioural tests (such as forced swim or treadmill) nor did we conduct any passive activity monitoring (such as voluntary wheel running or home-cage video tracking) which can be an alternative method to detect fatigue in mice [85], as this was outside the scope of our initial screen.

Meanwhile, genetic loss of *Macrod2* caused a hyperactive phenotype (as measured in LMA open field (large effect) and Y-maze preference test (medium effect)), which became more pronounced with age. The trend was also present in the bright field test however it wasn’t significant. One explanation for the diminished effect, as compared with the open field test could be that the testing time was much reduced (90 min vs. 2 or 5 min) and that there is more for mice to contend with in a Y-maze and bright field test setting. For example a choice of arms or a very bright light which mice find aversive [86] and can send them to sleep [87], confounding interpretation of results from a purely locomotor activity perspective. Nonetheless, the fact that the same trend of *Macrod2* KO mice moving more persists in a multitude of tests where total distance travelled was recorded is promising and further supports the notion that the *Macrod2* KO mice are hyperactive.

Interestingly, *Macrod2* KO hyperactivity was not correlated with a decrease in anxiety (less anxiety might mean more activity [88]) as determined by LMA open field and bright field. Additionally, since hyperactivity can be associated with decreased attention and increased impulsivity [89] it is interesting to note that short-term working memory and basic cognitive ability appears to be intact in these mice, as measured by Y-maze preference test. In the future, more in-depth cognitive and impulsivity testing, such as the radial arm maze, would provide further insight into the relevance of *Macrod2* to ADHD, autism and schizophrenia to which the genomic locus has been linked [45,46,47].

One confounding variable with regards to mouse age and test naivety is that the *Macrod2* cohort used repeated testing on the same mice at three months and 13 months. Experience and age can highly impact several aspects of mouse behaviour, including locomotion activity, willingness to explore and anxiety-like behaviours [90]. However, since there was no significant difference in central proportion exploration in either the LMA open field or bright field tests, with regards to the genotype, we hypothesise that the hyperactivity phenotype is genuine.

Most interestingly, the *Macrod2* KO hyperactivity phenotype was conversely paired with a bradykinesia type gait (slower, shorter steps as appears in Parkinson’s disease [75]). The unusual gait did not noticeably affect motor-coordination as there was a similar latency to fall in the accelerated rotarod test between genotypes and grip strength measurements were also not altered.

Macrod2 is primarily expressed in neurons [44] (and our unpublished data) and hyperactivity as well as gait disturbances are consistent with a neurological phenotype [91,92,93], although due to the constitutive nature of our KO model we cannot exclude a role of other organs or cell types. Neurological conditions, sleep and metabolism are delicately linked together and commonly disrupted in a plethora of human diseases [94,95,96]. Hence it is also interesting to note that *Macrod2* was identified as a novel sleep-related gene as part of a high-throughput screen in mice [58]. *Macrod2* KO mice slept significantly more during the dark, active phase versus controls; this corresponds well to the kind of sleep disruptions observed in ADHD (delayed sleep phase disorder, some 73–78% of children and adults with ADHD are affected [97]). Sleep disturbances are also common in autism (50–80% of children) [98]. The role of sleep in ADHD and mental health in general is so integral that some experts are calling for a rethink on how the diseases are perceived and thus treated [95,97].

With regards to the initial phenotyping of the *Macrod2* KO mice by the IMPC [57] and our phenotyping, where it overlaps, compares somewhat favourably. We could not confirm a smaller size however both us and the IMPC reported abnormal locomotor behaviour (KO mice moved further in a light-dark test). Since the IMPC do not usually keep mice beyond 16 weeks, it is highly likely they would not detect the increased hyperactivity with old age, or worsening gait. In terms of the IMPC metabolic defects, Lo Re et al. were unable to confirm a role for *Macrod2* in metabolism by glucose tolerance test, insulin tolerance test or in high fat diet induced obesity [72].

The lack of stronger phenotypes within these *Macrod1* and *Macrod2* KO mouse models is perhaps surprising given that genetic deficiency of other ADPr reversal enzymes, such as PARG (poly (ADP-ribose) glycohydrolase) can lead to early embryonic lethality in mice [99] or more severe neurological complications [17,20,22]. One potential explanation is redundancy between these two hydrolyses which might be expected given their similar biochemical activities [18,28,30]. On the other hand, quite distinct cellular localisations and tissue expression specificity (in the characterised cell/tissue models) between MACROD1 and MACROD2 would argue against a redundant/overlapping role in vivo, however the lack of strong phenotypes, indicates there must be sufficient residual activity provided by the remaining MAR and PAR hydrolases to compensate for their loss, at least outside conditions of stress. One benefit of a milder phenotype, is to provide a good proof of concept for the use of small molecule inhibitors against either protein should subsequent studies suggest a use for them (for example, anticancer therapies).

Generally, our newly observed behavioural phenotypes of *Macrod1* and *Macrod2* KO mice suggest that there is much to learn about these proteins, including roles beyond the ones most commonly studied in the ADPr field such as DNA damage response and genome stability. It will be interesting to extend these studies in the future to determine whether more complex forms of behaviour relevant to neurodevelopmental and neuropsychiatric disease are perturbed in these models. In terms of the underlying molecular mechanism, the physiological, in vivo, ADPr targets for MACROD1 and MACROD2 remain unknown or determined from in vitro studies using model ADPr substrates [18,25,26,27,28,29,30,33]. Experiments showing that KO Macrod1/2 orthologues in fungus *Neurospora crassa* affect levels of the sirtuin by-product O-acetyADP-ribose [30], suggest that physiological targets of Macrod1/2 enzymes could be beyond protein ADPr. Interestingly, very close homologues of MACROD1/2 are found in some viruses (usually referred to as viral macrodomains or mac1) including coronaviruses [100]. These viral mac1 domains are known to dampen interferon response by opposing activity of antiviral PARPs [9,101] and were shown to act on ADP-ribosylated RNAs in vitro suggestive of a possible role of MACROD1/2 in RNA modification [28]. Whilst the physiologically relevant targets of MACROD1/2 have yet to be determined, whether that be protein, RNA or DNA ADPr adducts or O-acetyADP-ribose, here we show that genetic deficiencies in either gene have physiological consequences relating to neuromuscular function and behaviour in vivo.

## Figures and Tables

**Table 1:** Behavioural testing cohort numbers and age at start of testing.

	*Macrod1* Cohort					*Macrod2* Cohort			
	MICE	WT	KO	TOTALS		MICE	WT	KO	TOTALS
12 months	Females	13	17	30	3 months	Females	20	17	37
	Males	19	15	34		Males	14	20	34
	TOTAL	32	32	64		TOTAL	34	37	71
18 months	Females	17	15	32	13 months	Females	16	16	32
	Males	13	12	25		Males	13	17	30
	TOTAL	30	27	57		TOTAL	29	33	62

**Table 2:** Descriptive statistics of weight data. Mean ± SEM is presented. Individual *N* number is included as well as *p*-values for all terms and interactions. *Macrod1* used a factorial-ANOVA. *Macrod2* used a factorial-ANOVA with repeated measures, hence the extra term Mouse Name. x = not an exact F-test.

Weight (g)	*Macrod1*			*Macrod2*		
	AGE	WT	*Macrod1* KO	AGE	WT	*Macrod1* KO
Female	12 months	31.96 ± 1.03	31.37 ± 1.13	3 months	21.33 ± 0.243	20.965 ± 0.321
		*n* = 13	*n* = 17		*n* = 20	*n* = 17
Male		43.50 ± 1.30	40.82 ± 1.24		28.857 ± 0.785	28.580 ± 0.494
		*n* = 19	*n* = 15		*n* = 14	*n* = 20
Female	18 months	39.21 ± 1.65	38.29 ± 1.29	13 months	29.525 ± 0.949	32.92 ± 1.25
		*n* = 17	*n* = 15		*n* = 16	*n* = 16
Male		50.83 ± 1.75	47.23 ± 1.88		42.31 ± 1.89	40.95 ± 1.89
		*n* = 13	*n* = 12		*n* = 13	*n* = 17
Genotype		F_(1,113)_ = 3.68, *p* = 0.058			F_(1,58)_ = 0.15, *p* = 0.701 x	
Sex		F_(1,113)_ = 104.70, * *p* < 0.001			F_(1,58)_ = 102.30, * *p* < 0.001 x	
Genotype*Sex		F_(1,113)_ = 1.39, *p* = 0.241			F_(1,58)_ = 1.66, *p* = 0.202 x	
Cohort		F_(1,113)_ = 47.23, * *p* < 0.001			F_(1,58)_ = 307.38, * *p* < 0.001	
Sex*Cohort		F_(1,113)_ = 0.01, *p* = 0.916			F_(1,58)_ = 4.12, * *p* = 0.047	
Genotype*Sex*Cohort		F_(1,113)_ = 0.02, *p* = 0.882			F_(1,58)_ = 3.29, *p* = 0.075	
Genotype*Cohort		F_(1,113)_ = 0.09, *p* = 0.759			F_(1,58)_ = 1.04, *p* = 0.312	
Mouse Name		N/A			F_(1,58)_ = 1.89, * *p* = 0.007	

**Table 3:** Descriptive statistics of data from *Macrod1* accelerated rotarod. Mean ± SEM is presented. Individual *N* number is included as well as *p*-values for all terms and interactions. Statistics was performed on all factors combined using a factorial-ANOVA, whereas separated by sex and age cohort comparisons used an ANCOVA (weight as a covariable). The difference between the means is presented alongside a Cohen’s *d*-score to demonstrate effect size.

Macrod1 Rotarod	AGE	Latency to Fall (s)				
		WT	*Macrod1* KO	Difference	*d*-Score	ANCOVA *p*-Value
Female	12 months	193.3 ± 12.7	146.0 ± 15.2	47.3	0.788	F_(1,27)_ = 14.53, * *p* = 0.005
		*n* = 13	*n* = 17			
Male		121.7 ± 10.7	117.1 ± 14.9	4.6	0.090	F_(1,22)_ = 0.53, *p* = 0.473
		*n* = 19	*n* = 15			
Female	18 months	123.8 ± 14.5	103.18 ± 9.52	20.62	0.408	F_(1,29)_ = 3.11, *p* = 0.089
		*n* = 17	*n* = 15			
Male		76.51 ± 5.05	78.92 ± 7.94	2.41	0.106	F_(1,22)_ = 0.15, *p* = 0.703
		*n* = 13	*n* = 12			
Genotype		F_(1,110)_ = 5.82, * *p* = 0.018				
Sex		F_(1,110)_ = 7.75, * *p* = 0.006				
Cohort		F_(1,110)_ = 3.66, *p* = 0.058				
Genotype*Sex		F_(1,110)_ = 0.01, *p* = 0.939				
Sex*Cohort		F_(1,110)_ = 2.36, *p* = 0.128				
Weight		F_(1,110)_ = 33.92, * *p* < 0.001				
Weight*Sex		F_(1,110)_ = 8.04, * *p* = 0.005				
Weight*Cohort		F_(1,110)_ = 2.50, *p* = 0.117				
Weight*Genotype		F_(1,110)_ = 4.13, * *p* = 0.045				
Genotype*Cohort		F_(1,110)_ = 0.01, *p* = 0.977				

**Table 4:** Descriptive statistics of data from *Macrod2* locomotor activity (LMA) open field. Mean ± SEM is presented. Individual *N* number is included as well as *p*-values for all terms and interactions. Statistics were performed on all factors combined using a factorial-ANOVA with repeated measures hence the term Mouse name. x = not an exact F-test. Separated by sex and age cohort comparisons used an ANCOVA (weight as a covariable). The difference between the means is presented alongside a Cohen’s d-score to demonstrate effect size.

Macrod2 Open Field	AGE	TOTAL Beam Breaks (90 min)				
		WT	*Macrod2* KO	Difference (s)	Cohen’s *d*	*p*-Value
Female	3 months	3698 ± 230	4367 ± 479	669	0.431	F_(1,33)_ = 1.20, *p* = 0.280
		*n* = 19	*n* = 17			
Male		3508 ± 173	4013 ± 212	505	0.585	F_(1,31)_ = 3.22, *p* = 0.082
		*n* = 14	*n* = 20			
Female	13 months	4403 ± 177	6727 ± 882	2324	0.840	F_(1,29)_ = 9.24, * *p* = 0.005
		*n* = 16	*n* = 16			
Male		3541 ± 205	4716 ± 338	1175	0.915	F_(1,27)_ = 6.68, * *p* = 0.015
		*n* = 13	*n* = 17			
Genotype		F_(1,55)_ = 12.14, * *p* = 0.001 x				
Sex		F_(1,55)_ = 0.54, *p* = 0.464 x				
Genotype*Sex		F_(1,55)_ = 1.31, *p* = 0.257 x				
Cohort		F_(1,55)_ = 1.92, *p* = 0.171				
Weight		F_(1,55)_ = 0.01, *p* = 0.917				
Weight*Sex		F_(1,55)_ = 0.24, *p* = 0.624				
Weight*Cohort		F_(1,55)_ = 0.74, *p* = 0.395				
Sex*Cohort		F_(1,55)_ = 1.28, *p* = 0.596				
Genotype*Cohort		F_(1,55)_ = 2.13, * *p* = 0.021				
Mouse name		F_(1,55)_ = 2.12, * *p* = 0.002				

**Table 5:** Descriptive statistics of distance travelled data from *Macrod2* distance travelled Y-maze preference test. Mean ± SEM is presented. Individual *N* number is included as well as *p*-values for all terms and interactions. Statistics were performed on all factors combined using a factorial-ANOVA with repeated measures hence the term Mouse name. x = not an exact F-test. Separated by sex and age cohort comparisons used an ANCOVA (weight as a covariable). The difference between the means is presented alongside a Cohen’s d-score to demonstrate effect size.

Macrod2 Y-Maze	AGE	Total Distance (m) (2 min)				
		WT	*Macrod2* KO	Difference (s)	Cohen’s d	*p*-Value
Female	3 months	6.328 ± 0.247	6.189 ± 0.490	0.139	0.094	F_(1,29)_ = 1.46, *p* = 0.505
		*n* = 17	*n* = 15			
Male		5.652 ± 0.284	6.524 ± 0.394	0.872	0.560	F_(1,30)_ = 2.38, *p* = 0.133
		*n* = 13	*n* = 20			
Female	13 months	3.743 ± 0.239	4.638 ± 0.452	0.895	0.560	F_(1,29)_ = 6.09, * *p* = 0.020
		*n* = 16	*n* = 16			
Male		3.659 ± 0.308	3.933 ± 0.308	0.274	0.237	F_(1,27)_ = 0.10, *p* = 0.759
		*n* = 13	*n* = 17			
Genotype		F_(1,51)_ = 5.74, * *p* = 0.019 x				
Sex		F_(1,51)_ = 0.46, *p* = 0.499 x				
Genotype*Sex		F_(1,51)_ = 0.07, *p* = 0.796 x				
Cohort		F_(1,51)_ = 0.03, *p* = 0.868				
Weight		F_(1,51)_ = 0.31, *p* = 0.581				
Weight*Sex		F_(1,51)_ = 0.57, *p* = 0.456				
Weight*Cohort		F_(1,51)_ = 0.05, *p* = 0.826				
Sex*Cohort		F_(1,51)_ = 0.26, *p* = 0.610				
Genotype*Cohort		F_(1,51)_ = 0.59, *p* = 0.447				
Mouse name		F_(1,51)_ = 0.95, *p* = 0.589				

**Table 6:** Descriptive statistics of gait velocity from the *Macrod2* catwalk gait analysis. Mean ± SEM is presented. Individual *N* number is included as well as *p*-values for all terms and interactions. Statistics were performed on all factors combined using a factorial-ANOVA with repeated measures hence the term Mouse name. x = not an exact F-test. Separated by sex and age cohort comparisons used an ANCOVA (weight as a covariable). The difference between the means is presented alongside a Cohen’s d-score to demonstrate effect size.

Macrod2 Catwalk	AGE	Gait Velocity (cm/s)				
		WT	*Macrod2* KO	Difference (cm/s)	Cohen’s *d*	*p*-Value
Female	3 months	27.586 ± 0.926	25.84 ± 1.36	1.746	0.358	F_(1,34)_ = 1.15, *p* = 0.290
		*n* = 20	*n* = 17			
Male		27.339 ± 0.818	22.917 ± 0.751	4.422	1.188	F_(1,27)_ = 15.09, * *p* = 0.001
		*n* = 13	*n* = 17			
Female	13 months	21.707 ± 0.805	19.701 ± 0.717	2.006	0.633	F_(1,29)_ = 4.33, * *p* = 0.046
		*n* = 16	*n* = 16			
Male		22.192 ± 0.800	19.263 ± 0.831	2.929	0.842	F_(1,27)_ = 7.98, * *p* = 0.009
		*n* = 13	*n* = 17			
Genotype		F_(1,54)_ = 13.69, * *p* < 0.001 x				
Sex		F_(1,54)_ = 0.97, *p* = 0.330 x				
Genotype*Sex		F_(1,54)_ = 3.31, *p* = 0.073 x				
Cohort		F_(1,54)_ = 0.93, *p* = 0.339				
Weight		F_(1,54)_ = 2.89, *p* = 0.095				
Weight*Sex		F_(1,54)_ = 0.39, *p* = 0.535				
Weight*Cohort		F_(1,54)_ = 0.54, *p* = 0.467				
Sex*Cohort		F_(1,54)_ = 0.14, *p* = 0.708				
Genotype*Cohort		F_(1,54)_ = 0.35, *p* = 0.558				
Mouse Name		F_(1,54)_ = 1.48, *p* = 0.069				

**Table 7:** Descriptive statistics of SQRT gait cadence from the *Macrod2* catwalk gait analysis. SQRT = square root transformed. Mean ± SEM is presented. Individual *N* number is included as well as *p*-values for all terms and interactions. Statistics were performed on all factors combined using a factorial-ANOVA with repeated measures hence the term Mouse Name. x = not an exact F-test. Separated by sex and age cohort comparisons used an ANCOVA (weight as a covariable). The difference between the means is presented alongside a Cohen’s d-score to demonstrate effect size.

Macrod2 Catwalk	AGE	SQRT Gait Cadence (Steps/Minute)				
		WT	*Macrod2* KO	Difference	Cohen’s *d*	*p*-Value
Female	3 months	4.1927 ± 0.0449 *n* = 20	4.1121 ± 0.0606 *n* = 17	0.0806	0.358	F_(1,34)_ = 1.16, *p* = 0.289
Male		4.0171 ± 0.0530 *n* = 13	3.8959 ± 0.0461 *n* = 17	0.1212	0.616	F_(1,27)_ = 3.00, *p* = 0.095
Female	13 months	3.7722 ± 0.0521 *n* = 16	3.6424 ± 0.0429 *n* = 16	0.1298	0.653	F_(1,29)_ = 2.20, *p* = 0.149
Male		3.7295 ± 0.0614 *n* = 13	3.6475 ± 0.0586 *n* = 17	0.082	0.352	F_(1,27)_ = 1.71, *p* = 0.202
Genotype		F_(1,54)_ = 5.15, * *p* = 0.026 x				
Sex		F_(1,54)_ = 0.59, *p* = 0.445 x				
Genotype*Sex		F_(1,54)_ = 0.52, *p* = 0.473 x				
Cohort		F_(1,54)_ = 0.46, *p* = 0.499				
Weight		F_(1,54)_ = 4.47, * *p* = 0.039				
Weight*Sex		F_(1,54)_ = 0.20, *p* = 0.659				
Weight*Cohort		F_(1,54)_ = 0.20, *p* = 0.658				
Sex*Cohort		F_(1,54)_ = 0.96, *p* = 0.330				
Genotype*Cohort		F_(1,54)_ = 0.01, *p* = 0.903				
Mouse Name		F_(1,54)_ = 1.92, * *p* = 0.007				

**Table 8:** Descriptive statistics of forefoot swing speed from the *Macrod2* catwalk gait analysis. Mean ± SEM is presented. Individual *N* number is included as well as *p*-values for all terms and interactions. Statistics were performed on all factors combined using a factorial-ANOVA with repeated measures hence the term Mouse Name. x = not an exact F-test. Separated by sex and age cohort comparisons used an ANCOVA (weight as a covariable). The difference between the means is presented alongside a Cohen’s d-score to demonstrate effect size.

Macrod2 Catwalk	Forefoot Swing Speed (cm/s)					
	AGE	WT	*Macrod2* KO	Difference (cm/s)	Cohen’s *d*	*p*-Value
Female	3 months	60.67 ± 1.43 *n* = 20	57.92 ± 2.68 *n* = 17	2.75	0.311	F_(1,34)_ = 0.639, *p* = 0.359
Male		63.32 ± 1.45 *n* = 13	56.22 ± 1.51 *n* = 17	7.1	1.053	F_(1,27)_ = 10.82, * *p* = 0.003
Female	13 months	49.57 ± 1.43 *n* = 16	48.41 ± 1.51 *n* = 16	1.16	0.200	F_(1,29)_ = 1.86, *p* = 0.183
Male		54.37 ± 1.93 *n* = 13	48.07 ± 1.38 *n* = 17	6.3	0.912	F_(1,27)_ = 7.26, * *p* = 0.012
Genotype		F_(1,54)_ = 11.93, * *p* = 0.001 x				
Sex		F_(1,54)_ = 1.65, *p* = 0.204 x				
Genotype*Sex		F_(1,54)_ = 3.31, *p* = 0.073 x				
Cohort		F_(1,54)_ = 1.22, *p* = 0.275				
Weight		F_(1,54)_ = 1.52, *p* = 0.224				
Weight*Sex		F_(1,54)_ = 0.81, *p* = 0.373				
Weight*Cohort		F_(1,54)_ = 0.57, *p* = 0.452				
Sex*Cohort		F_(1,54)_ = 0.07, *p* = 0.790				
Genotype*Cohort		F_(1,54)_ = 0.14, *p* = 0.706				
Mouse Name		F_(1,54)_ = 1.33, *p* = 0.143				

**Table 9:** Descriptive statistics of forefoot stride length from the *Macrod2* catwalk gait analysis. Mean ± SEM is presented. Individual *N* number is included as well as *p*-values for all terms and interactions. Statistics were performed on all factors combined using a factorial-ANOVA with repeated measures hence the term Mouse Name. x = not an exact F-test. Separated by sex and age cohort comparisons used an ANCOVA (weight as a covariable). The difference between the means is presented alongside a Cohen’s d-score to demonstrate effect size.

Macrod2 Catwalk	AGE	Forefoot Stride Length (cm)				
		WT	Macrod2 KO	Difference (cm/s)	Cohen’s *d*	*p*-Value
Female	3 months	6.166 ± 0.113 *n* = 20	5.934 ± 0.200 *n* = 17	0.232	0.345	F_(1,34)_ = 1.07, *p* = 0.309
Male		6.755 ± 0.118 *n* = 13	5.989 ± 0.100 *n* = 17	0.766	1.361	F_(1,27)_ = 23.85, * *p* > 0.001
Female	13 months	6.078 ± 0.149 *n* = 16	5.838 ± 0.145 *n* = 16	0.240	0.406	F_(1,29)_ = 3.63, *p* = 0.067
Male		6.3687 ± 0.0804 *n* = 13	5.816 ± 0.134 *n* = 17	0.5527	1.045	F_(1,27)_ = 10.32, * *p* = 0.003
Genotype		F_(1,54)_ = 18.48, * *p* < 0.001 x				
Sex		F_(1,54)_ = 2.28, *p* = 0.137 x				
Genotype*Sex		F_(1,54)_ = 4.70, * *p* = 0.033 x				
Cohort		F_(1,54)_ = 0.41, *p* = 0.526				
Weight		F_(1,54)_ = 0.77, *p* = 0.383				
Weight*Sex		F_(1,54)_ = 1.23, *p* = 0.271				
Weight*Cohort		F_(1,54)_ = 0.40, *p* = 0.529				
Sex*Cohort		F_(1,54)_ = 0.00, *p* = 0.988				
Genotype*Cohort		F_(1,54)_ = 0.14, *p* = 0.715				
Mouse Name		F_(1,54)_ = 1.53, *p* = 0.054				

## Data Availability

The data presented in this study are available in the article or Supplementary Materials.

## Supplementary Materials

The following are available online at https://www.mdpi.com/2073-4409/10/2/368/s1, Table S1: Descriptive statistics of data from *Macrod2* accelerated rotarod. Figure S1: *Macrod2* KO is not associated with reduced fall latency on the rotarod. Table S2: Descriptive statistics of central proportion from *Macrod2* open field. Figure S2: *Macrod2* KO is not associated with anxiety in LMA open field. Table S3: Descriptive statistics of data from *Macrod1* LMA open field. Figure S3: Macrod1 KO is not associated with changes in locomotor activity or central proportion in LMA open field. Table S4: Descriptive statistics of data from *Macrod2* LMA bright field. Figure S4: *Macrod2* KO is not associated with changes in locomotor activity or central proportion in LMA bright field. Table S5: Descriptive statistics of data from *Macrod1* LMA bright field. Figure S5: *Macrod1* KO is not associated with changes in locomotor activity or central proportion in LMA bright field. Table S6: Descriptive statistics of preference ratio from *Macrod2* Y-maze preference test. Figure S6: *Macrod2* KO is not associated with changes in short term memory and attention. Table S7: Descriptive statistics of data from *Macrod1* LMA bright field. Table S8: Descriptive statistics of distance travelled data from *Macrod1* distance travelled Y-maze preference test. Figure S7: *Macrod1* KO is not associated with changes in short term memory and attention. Table S9: Descriptive statistics of SQRT-fore and hind paw intensity for *Macrod1* from the catwalk gait analysis. Figure S8: *Macrod1* KO is not associated with changes fore or hind paw foot pressure. Table S10: Descriptive statistics of gait velocity and SQRT gait cadence for *Macrod1* from the catwalk gait analysis. Table S11: Descriptive statistics of forefoot swing speed and forefoot stride length for *Macrod1* from the catwalk gait analysis. Figure S9: *Macrod1* KO is not associated an altered gait. Table S12: Descriptive statistics of SQRT fore and hind paw intensity for *Macrod2* from the catwalk gait analysis. Figure S10: *Macrod2* KO is not associated with changes in fore or hind paw foot pressure. Table S13: Descriptive statistics of *Macrod1* grip strength. Figure S11: *Macrod1* KO is not associated altered grip strength. Table S14: Descriptive statistics of *Macrod2* grip strength. Figure S12: *Macrod2* KO is not associated altered grip strength.
