# Supplementary material for: Behavioural Characterisation of Macrod1 and Macrod2 Knockout Mice
Source: Cells. 2021 Feb 10;10(2):368. doi: 10.3390/cells10020368 (PMC7916507; doi:10.3390/cells10020368)
Supplement: Supplementary file 1 [file cells-10-00368-s001.pdf]

Supplemental information

Table S1: Descriptive statistics of data from *Macrod2* accelerated rotarod. Mean ± SEM is presented. Individual N number is included as well as p-values for all terms and interactions. *Macrod2* used a factorial-ANOVA with repeated measures, hence the term Mouse name. x = not an exact F-test.

***Macrod2* Rotarod**	***Latency to fall (s)***		
	AGE	WT	*Macrod2* KO
Female	3 months	196.3 ± 13.2<br>N=20	201.1 ± 11.3<br>N=17
Male	3 months	191.0 ± 12.9<br>N=14	155.70 ± 9.74<br>N=20
Female	13 months	207.6 ± 12.7<br>N=16	193.0 ± 12.5<br>N=16
Male	13 months	170.3 ± 15.5<br>N=13	186.9 ± 18.3<br>N=17
Genotype	$F_{(1,56)}$=0.97, p=0.327 x		
Sex	$F_{(1,56)}$=1.14, p=0.290 x		
Genotype*Sex	$F_{(1,56)}$=1.13, p=0.291 x		
Cohort	$F_{(1,56)}$=0.29, p=0.594		
Weight	$F_{(1,56)}$=18.49, p=0.006		
Weight*Sex	$F_{(1,56)}$=0.28, p=0.597		
Weight*Cohort	$F_{(1,56)}$=0.18, p=0.675		
Sex*Cohort	$F_{(1,56)}$=0.34, p=0.562		
Genotype*Cohort	$F_{(1,56)}$=1.11, p=0.296		
Mouse name	$F_{(1,56)}$=2.05, *p=0.003		

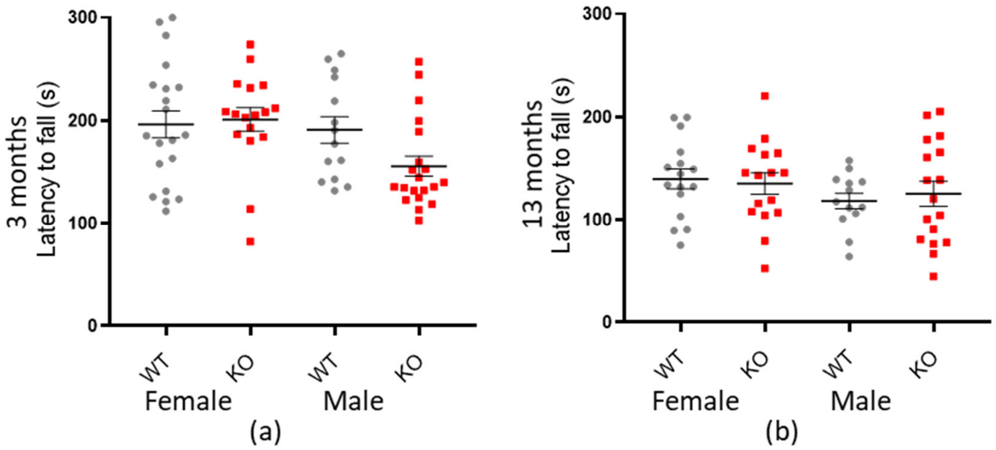

Figure S1: *Macrod2* KO is not associated with reduced fall latency on the rotarod. Graphs show individual values dot plot, WT shown in grey circles and Macrod2 KO in red squares. Error bars are the SEM. Graphs are as follows **a-b)** Macrod2 3 and 13 months.

Table S2: Descriptive statistics of central proportion from *Macrod2* open field. Mean ± SEM is presented. Individual N number is included as well as p-values for all terms and interactions. *Macrod2* used a factorial-ANOVA with repeated measures, hence the term Mouse name. x = not an exact F-test.

Macrod2 Open Field	***Central %***		
	AGE	WT	*Macrod2* KO
Female	3 months	35.41 ± 1.59 N=19	39.35 ± 2.28 N=17
Male	3 months	39.61 ± 1.36 N=14	43.40 ± 1.80 N=20
Female	13 months	35.47 ± 1.55 N=16	37.71 ± 1.81 N=16
Male	13 months	40.11 ± 1.19 N=13	39.73 ± 1.74 N=17
Genotype		$F_{(1,55)}$=2.79, p=0.099 x	
Sex		$F_{(1,55)}$=0.23, p=0.635 x	
Genotype*Sex		$F_{(1,55)}$=0.11, p=0.737 x	
Cohort		$F_{(1,55)}$=0.46, p=0.501	
Weight		$F_{(1,55)}$=0.36, p=0.554	
Weight*Sex		$F_{(1,55)}$=0.38, p=0.538	
Weight*Cohort		$F_{(1,55)}$=0.60, p=0.441	
Sex*Cohort		$F_{(1,55)}$=0.01, p=0.917	
Genotype*Cohort		$F_{(1,55)}$=1.73, p=0.194	
Mouse name		$F_{(1,55)}$=1.68, *p=0.024	

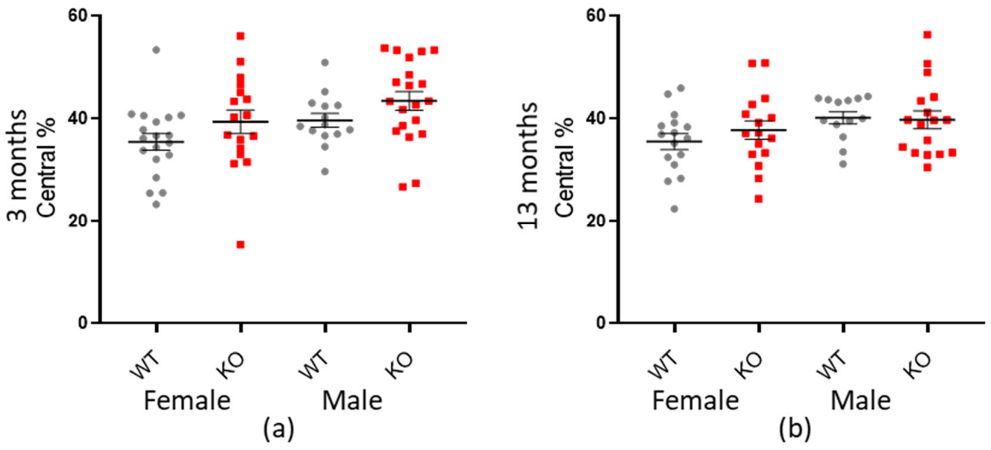

Figure S2: Macrod2 KO is not associated with anxiety in LMA Open Field. Graphs show individual values dot plot, WT shown in grey circles and Macrod2 KO in red squares. Error bars are the SEM. Graphs are as follows **a-b)** Macrod2 3 and 13 months.

Table S3: Descriptive statistics of data from *Macrod1* LMA Open Field. Mean ± SEM is presented. Individual N number is included as well as p-values for all terms and interactions. *Macrod1* used a factorial-ANOVA.

Macrod1* *Open Field		**Total distance (beam breaks)**		**Central %**	
	AGE	WT	*Macrod1* KO	WT	*Macrod1* KO
Female	12 months	4704 ± 321 N=13	4889 ± 301 N=17	38.32 ± 1.14 N=13	39.51 ± 1.72 N=17
Male	12 months	4530 ± 374 N=12	5161 ± 483 N=12	36.46 ± 1.49 N=12	37.74 ± 1.57 N=12
Female	18 months	5660 ± 251 N=17	5111 ± 357 N=14	37.65 ± 1.05 N=17	37.93 ± 1.29 N=14
Male	18 months	4123 ± 247 N=14	4074 ± 295 N=12	40.52 ± 1.54 N=14	41.50 ± 1.94 N=12
Genotype		$F_{(1,100)}$=0.64, p=0.427		$F_{(1,100)}$=0.42, p=0.517	
Sex		$F_{(1,100)}$=0.05, p=0.826		$F_{(1,100)}$=1.16, p=0.284	
Genotype*Sex		$F_{(1,100)}$=0.00, p=0.981		$F_{(1,100)}$=0.34, p=0.561	
Cohort		$F_{(1,100)}$=0.55, p=0.461		$F_{(1,100)}$=0.95, p=0.332	
Weight		$F_{(1,100)}$=2.67, p=0.105		$F_{(1,100)}$=1.85, p=0.176	
Weight*Sex		$F_{(1,100)}$=0.01, p=0.921		$F_{(1,100)}$=0.64, p=0.426	
Weight*Cohort		$F_{(1,100)}$=0.41, p=0.524		$F_{(1,100)}$=0.34, p=0.561	
Weight*Genotype		$F_{(1,100)}$=0.63, p=0.430		$F_{(1,100)}$=0.53, p=0.467	
Sex*Cohort		$F_{(1,100)}$=2.14, p=0.146		$F_{(1,100)}$=0.92, p=0.340	
Genotype*Cohort		$F_{(1,100)}$=3.45, p=0.066		$F_{(1,100)}$=0.24, p=0.624	

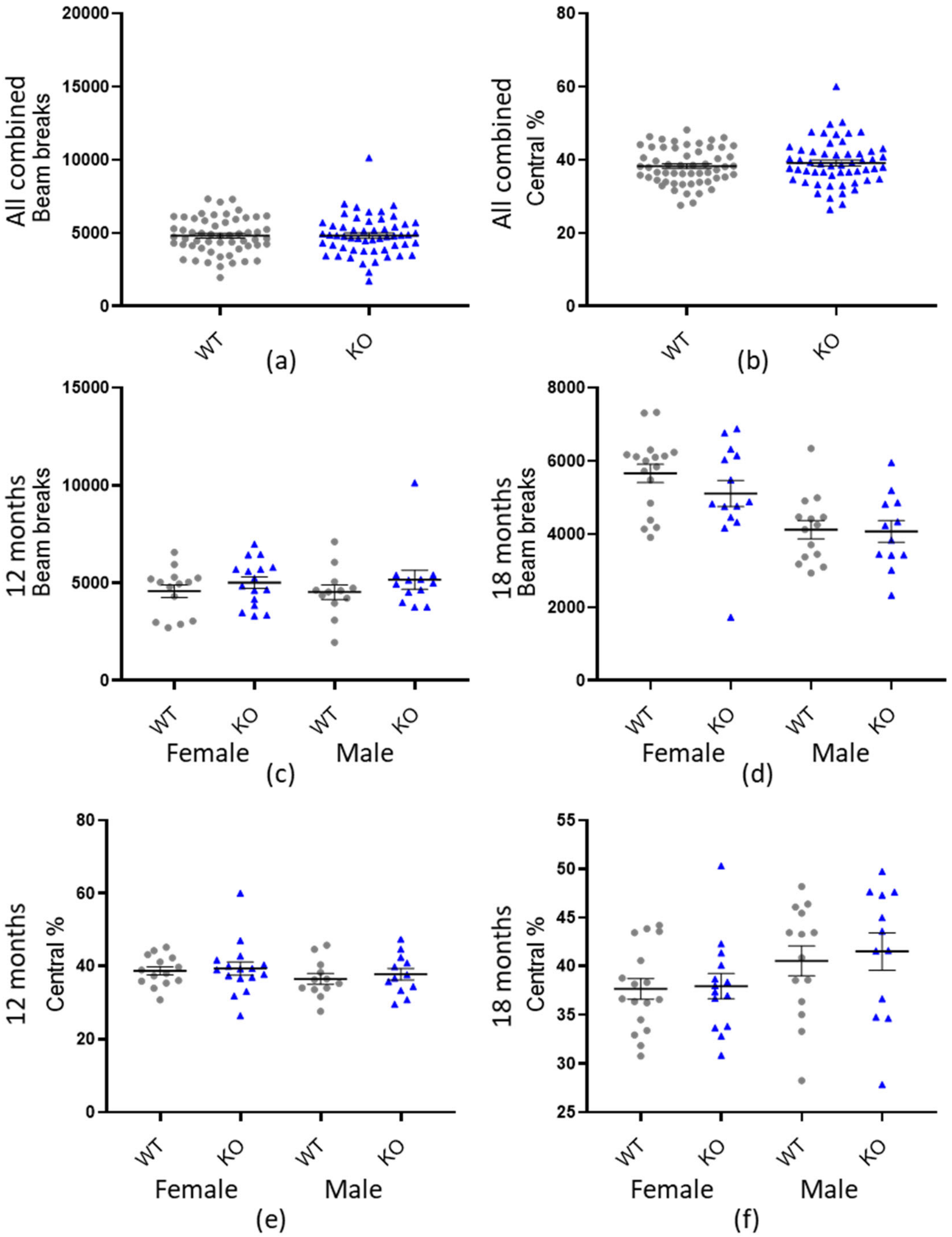

Figure S3: *Macrod1* KO is not associated with changes in locomotor activity or central proportion in LMA open field. Graphs show individual values dot plot, WT shown in grey circles and Macrod1 KO in blue triangles. Error bars are the SEM. Graphs are as follows **a, c-d)** Macrod1 LMA total beam breaks all combined, 12 and 18 months. **b, e-f)** Macrod1 LMA central proportion (%) all combined, 12 and 18 months.

Table S4: Descriptive statistics of data from *Macrod2* LMA Bright Field. Mean ± SEM is presented. Individual N number is included as well as p-values for all terms and interactions. *Macrod2* used a factorial-ANOVA with repeated measures, hence the term Mouse name. x = not an exact F-test.

***Macrod2* Bright Field**		***Total distance (m)***		***Central (s)***	
	AGE	WT	*Macrod2* KO	WT	*Macrod2* KO
Female	3 months	26.06 ± 1.00<br>N=20	28.44 ± 1.48<br>N=17	8.18 ± 1.23<br>N=20	6.153 ± 0.802<br>N=17
Male	3 months	21.75 ± 1.02<br>N=14	24.69 ± 1.27<br>N=20	12.51 ± 1.02<br>N=14	9.73 ± 1.09<br>N=20
Female	13 months	31.75 ± 2.00<br>N=15	32.77 ± 2.64<br>N=16	5.595 ± 0.869<br>N=15	9.37 ± 1.22<br>N=16
Male	13 months	21.88 ± 1.48<br>N=13	26.83 ± 2.04<br>N=17	5.778 ± 0.954<br>N=13	5.595 ± 0.974<br>N=17
Genotype		$F_{(1,55)}$=3.33, p=0.072 x		$F_{(1,55)}$=0.00, p=0.955 x	
Sex		$F_{(1,55)}$=1.85, p=0.179 x		$F_{(1,55)}$=4.05, p=0.049 x	
Genotype*Sex		$F_{(1,55)}$=0.16, p=0.692 x		$F_{(1,55)}$=3.44, p=0.067 x	
Cohort		$F_{(1,55)}$=0.47, p=0.497		$F_{(1,55)}$=1.18, p=0.283	
Weight		$F_{(1,55)}$=0.03, p=0.870		$F_{(1,55)}$=0.00, p=0.955	
Weight*Sex		$F_{(1,55)}$=3.73, p=0.059		$F_{(1,55)}$=3.27, p=0.076	
Weight*Cohort		$F_{(1,55)}$=0.08, p=0.781		$F_{(1,55)}$=1.08, p=0.303	
Sex*Cohort		$F_{(1,55)}$=0.95, p=0.334		$F_{(1,55)}$=0.97, p=0.330	
Genotype*Cohort		$F_{(1,55)}$=0.04, p=0.847		$F_{(1,55)}$=7.71, *p=0.008	
Mouse name		$F_{(1,55)}$=2.10, *p=0.003		$F_{(1,55)}$=0.96, p=0.570	

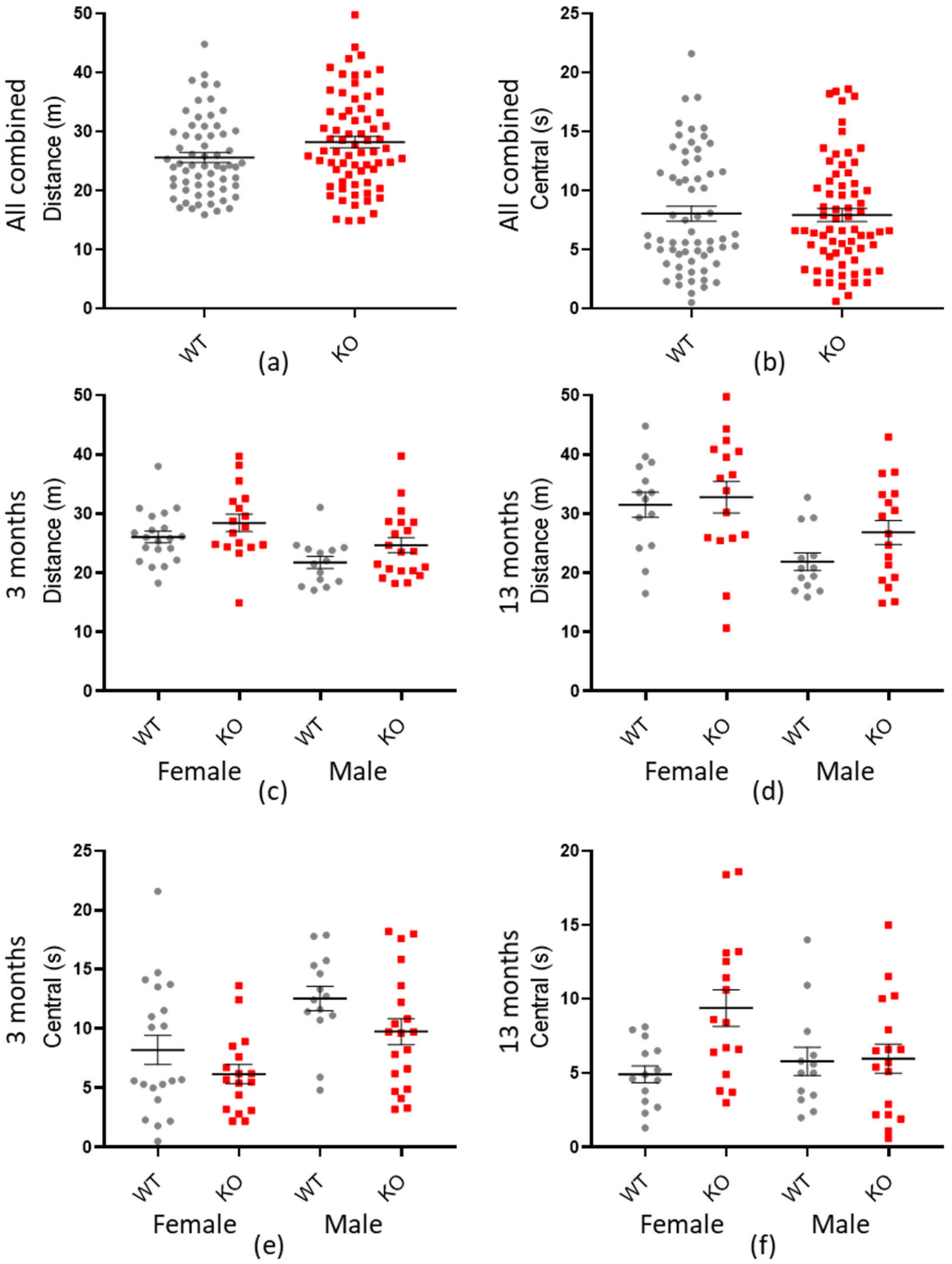

Figure S4: *Macrod2* KO is not associated with changes in locomotor activity or central proportion in LMA bright field. Graphs show individual values dot plot, WT shown in grey circles and Macrod2 KO in red squares. Error bars are the SEM. Graphs are as follows **a, c-d)** total distance travelled (m) all combined, 3 and 13 months. **b, e-f)** central proportion (s) all combined, 3 and 13 months.

Table S5: Descriptive statistics of data from *Macrod1* LMA Bright Field. Mean ± SEM is presented. Individual N number is included as well as p-values for all terms and interactions. Macrod1 used a factorial-ANOVA.

Macrod1* *Bright Field		**Total distance (m)**		**Central (s)**	
	AGE	WT	*Macrod1* KO	WT	*Macrod1* KO
Female	12 months	17.81 ± 1.29<br>N=10	18.62 ± 1.26<br>N=17	5.680 ± 0.915<br>N=10	4.724 ± 0.730<br>N=17
Male	12 months	17.41 ± 2.40<br>N=11	18.26 ± 2.09<br>N=12	6.16 ± 1.93<br>N=11	7.79 ± 1.48<br>N=12
Female	18 months	19.96 ± 1.26<br>N=17	23.46 ± 1.60<br>N=14	3.765 ± 0.589<br>N=17	4.314 ± 0.584<br>N=14
Male	18 months	16.21 ± 1.50<br>N=10	14.83 ± 1.67<br>N=11	4.96 ± 1.66<br>N=10	4.03 ± 1.10<br>N=11
Genotype		$F_{(1,91)}$=0.34, p=0.559		$F_{(1,91)}$=1.57, p=0.214	
Sex		$F_{(1,91)}$=1.00, p=0.320		$F_{(1,91)}$=0.16, p=0.687	
Genotype*Sex		$F_{(1,91)}$=2.35, p=0.129		$F_{(1,91)}$=0.64, p=0.427	
Cohort		$F_{(1,91)}$=3.27, p=0.074		$F_{(1,91)}$=7.16, *p=0.009	
Weight		$F_{(1,91)}$=5.49, *p=0.021		$F_{(1,91)}$=4.02, *p=0.048	
Weight*Sex		$F_{(1,91)}$=1.10, p=0.297		$F_{(1,91)}$=0.01, p=0.914	
Weight*Cohort		$F_{(1,91)}$=4.19, *p=0.043		$F_{(1,91)}$=6.30, *p=0.014	
Weight*Genotype		$F_{(1,91)}$=0.40, p=0.529		$F_{(1,91)}$=1.51, p=0.222	
Sex*Cohort		$F_{(1,91)}$=6.78, *p=0.011		$F_{(1,91)}$=5.23, *p=0.024	
Genotype*Cohort		$F_{(1,91)}$=0.04, p=0.849		$F_{(1,91)}$=0.17, p=0.680	

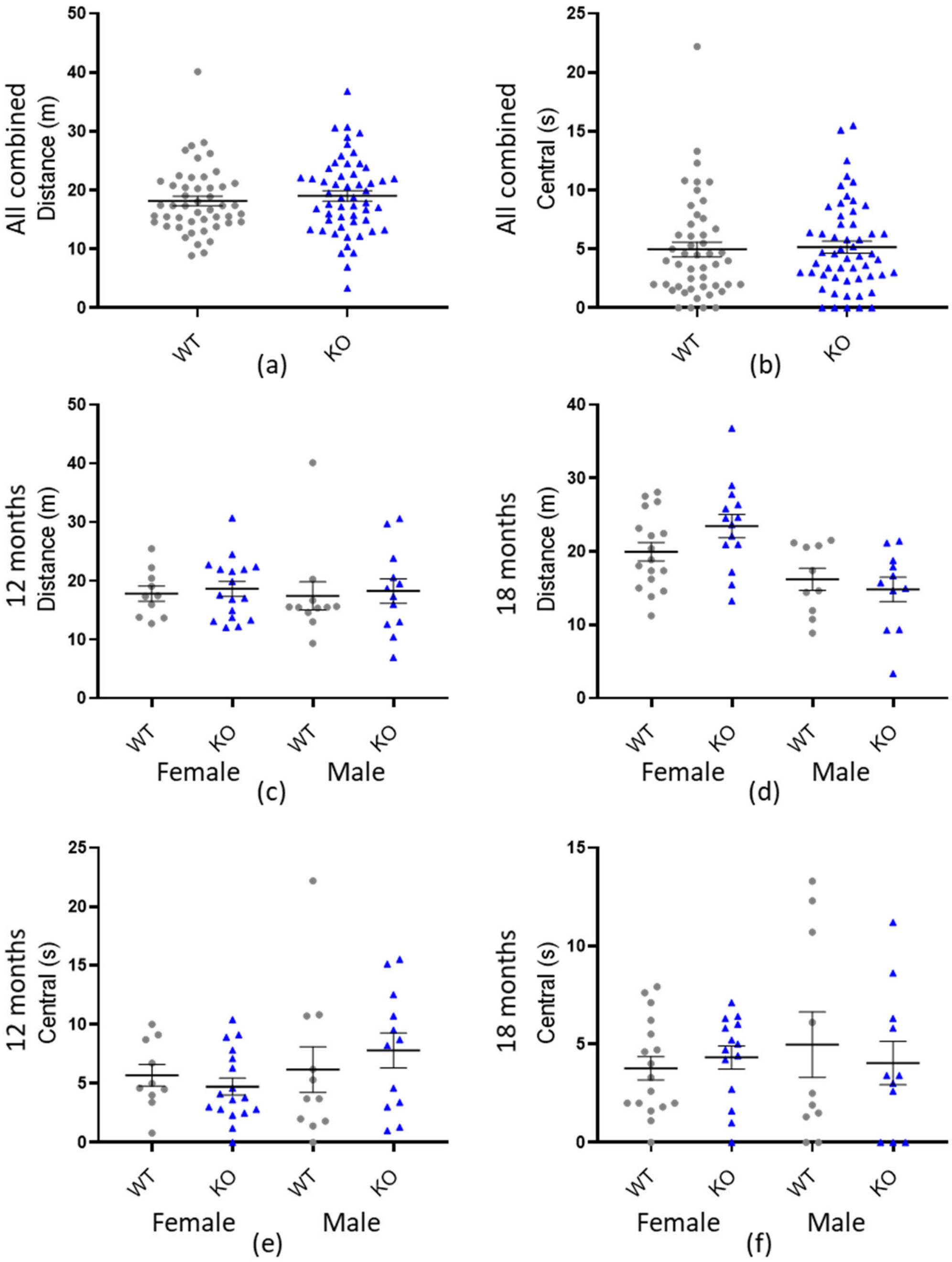

Figure S5: *Macrod1* KO is not associated with changes in locomotor activity or central proportion in LMA bright field. Graphs show individual values dot plot, WT shown in grey circles and Macrod1 KO in blue triangles. Error bars are the SEM. Graphs are as follows **a, c-d)** total distance travelled (m), all data combined, 12 and 18 months. **b, e-f)** central proportion (s) all combined, 12 and 18 months.

Table S6: Descriptive statistics of preference ratio from *Macrod2* Y-maze preference test. Mean ± SEM is presented. Individual N number is included as well as p-values for all terms and interactions. *Macrod2* used a factorial-ANOVA with repeated measures, hence the term Mouse name. x = not an exact F-test.

Macrod2 Y-maze	***Preference ratio***		
	AGE	WT	*Macrod2* KO
Female	3 months	0.7169 ± 0.0359 N=17	0.6886 ± 0.0376 N=15
Male	3 months	0.7628 ± 0.0251 N=13	0.6594 ± 0.0223 N=20
Female	13 months	0.6006 ± 0.0528 N=16	0.6075 ± 0.0527 N=16
Male	13 months	0.6127± 0.0614 N=13	0.6107 ± 0.0563 N=17
Genotype		$F_{(1,51)}$=0.41, p=0.522 x	
Sex		$F_{(1,51)}$=0.31, p=0.580 x	
Genotype*Sex		$F_{(1,51)}$=0.65, p=0.423 x	
Cohort		$F_{(1,51)}$=0.35, p=0.557	
Weight		$F_{(1,51)}$=0.07, p=0.787	
Weight*Sex		$F_{(1,51)}$=0.40, p=0.531	
Weight*Cohort		$F_{(1,51)}$=0.11, p=0.737	
Sex*Cohort		$F_{(1,51)}$=0.38, p=0.543	
Genotype*Cohort		$F_{(1,51)}$=1.09, p=0.301	
Mouse name		$F_{(1,51)}$=1.05, p=0.436	

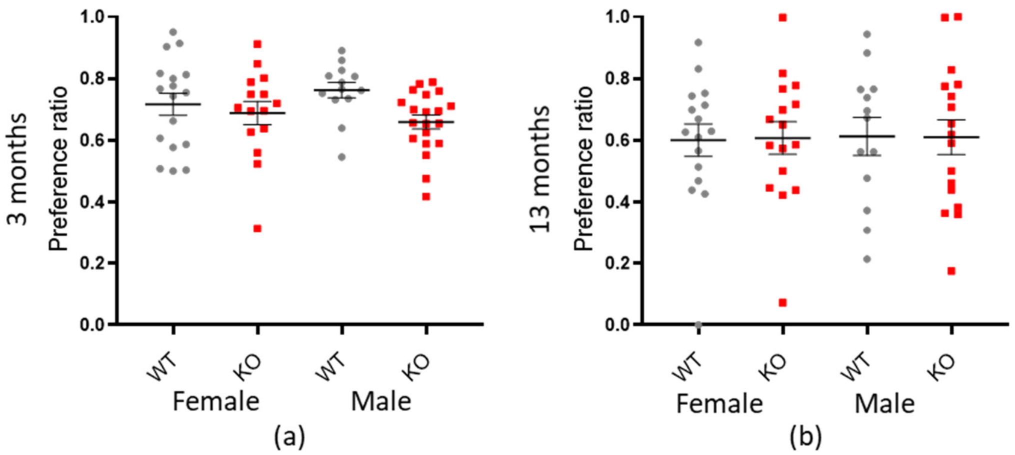

Figure S6: *Macrod2* KO is not associated with changes in short term memory and attention. Graphs show individual values dot plot, WT shown in grey circles and Macrod2 KO in red squares. Error bars are the SEM. Graphs are as follows **a-b)** Preference ratio (from y-maze preference test) at 3 and 13 months.

Table S7: Descriptive statistics of data from *Macrod1* LMA Bright Field. Mean ± SEM is presented. Individual N number is included as well as p-values for all terms and interactions. *Macrod1* used a factorial-ANOVA.

Macrod1* *Y-maze		***Preference ratio***		***Distance travelled (m)***	
	AGE	WT	*Macrod1* KO	WT	*Macrod1* KO
Female	12 months	0.6980 ± 0.0371 N=13	0.5785 ± 0.0571 N=17	6.113 ± 0.210 N=13	5.071 ± 0.396 N=17
Male	12 months	0.6845 ± 0.0432 N=18	0.6689 ± 0.0507 N=13	4.953 ± 0.335 N=18	5.209 ± 0.299 N=13
Female	18 months	0.6703 ± 0.0526 N=17	0.6294 ± 0.0638 N=15	5.036 ± 0.510 N=17	4.996 ± 0.335 N=15
Male	18 months	0.5626 ± 0.0858 N=13	0.4771 ± 0.0662 N=12	3.573 ± 0.413 N=13	3.677 ± 0.440 N=12
Genotype		$F_{(1,107)}$=0.06, p=0.805		$F_{(1,107)}$=9.30, *p=0.003	
Sex		$F_{(1,107)}$=1.67, p=0.200		$F_{(1,107)}$=0.49, p=0.485	
Genotype*Sex		$F_{(1,107)}$=0.01, p=0.910		$F_{(1,107)}$=1.06, p=0.306	
Cohort		$F_{(1,107)}$=0.11, p=0.735		$F_{(1,107)}$=0.26, p=0.612	
Weight		$F_{(1,107)}$=0.53, p=0.468		$F_{(1,107)}$=2.50, p=0.117	
Weight*Sex		$F_{(1,107)}$=2.36, p=0.127		$F_{(1,107)}$=0.21, p=0.652	
Weight*Cohort		$F_{(1,107)}$=0.40, p=0.528		$F_{(1,107)}$=0.02, p=0.879	
Weight*Genotype		$F_{(1,107)}$=0.00, p=0.966		$F_{(1,107)}$=8.71, *p=0.004	
Sex*Cohort		$F_{(1,107)}$=0.07, p=0.790		$F_{(1,107)}$=1.87, p=0.175	
Genotype*Cohort		$F_{(1,107)}$=0.00, p=0.995		$F_{(1,107)}$=0.17, p=0.385	

Table S8: Descriptive statistics of distance travelled data from *Macrod1* distance travelled Y-maze preference test. Mean ± SEM is presented. Individual N number is included as well as p-values for all terms and interactions. Statistics was performed on all factors combined using a factorial-ANOVA. Whereas, separated by sex and age cohort comparisons used an ANCOVA (weight as a co-variable). The difference between the means is presented alongside a Cohen's d-score to demonstrate effect size.

Macrod1 Y-maze	AGE	Total distance (m)				
		WT	*Macrod1* KO	Difference (m)	Cohen's d	*p-value*
Female	12 months	6.113 ± 0.210 N=13	5.071 ± 0.396 N=17	1.042	0.740	$F_{(1,27)}$=4.31, *p=0.047
Male	12 months	4.953 ± 0.335 N=18	5.209 ± 0.299 N=13	0.256	0.201	$F_{(1,28)}$=0.03, p=0.869
Female	18 months	5.036 ± 0.510 N=17	4.996 ± 0.335 N=15	0.04	0.023	$F_{(1,29)}$=0.07, p=0.791
Male	18 months	3.573 ± 0.413 N=13	3.677 ± 0.440 N=12	0.104	0.071	$F_{(1,22)}$=0.00, p=0.958

Genotype	$F_{(1,107)}$=9.30, *p=0.003
Sex	$F_{(1,107)}$=0.49, p=0.485
Genotype*Sex	$F_{(1,107)}$=1.06, p=0.306
Cohort	$F_{(1,107)}$=0.26, p=0.612
Weight	$F_{(1,107)}$=2.50, p=0.117
Weight*Sex	$F_{(1,107)}$=0.21, p=0.652
Weight*Cohort	$F_{(1,107)}$=0.02, p=0.879
Weight*Genotype	$F_{(1,107)}$=8.71, *p=0.004
Sex*Cohort	$F_{(1,107)}$=1.87, p=0.175
Genotype*Cohort	$F_{(1,107)}$=0.17, p=0.385

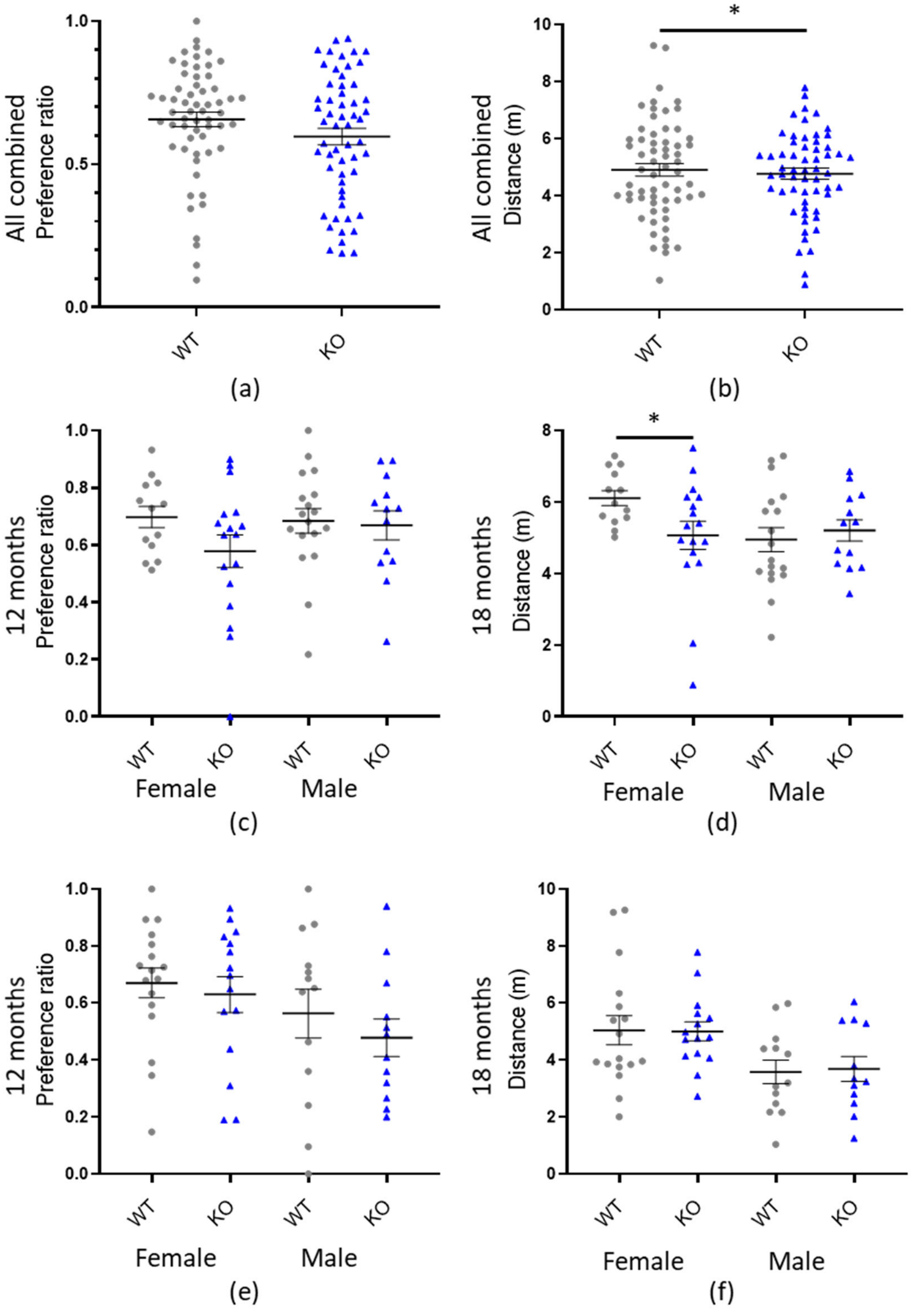

Figure S7: *Macrod1* KO is not associated with changes in short term memory and attention. Results from the Y-maze preference test. Graphs show individual values dot plot, WT shown in grey circles and Macrod1 KO in blue triangles. Error bars are the SEM. Graphs are as follows **a, c-d)** preference ratio, all data combined, 12 and 18 months. **b, e-f)** total distance travelled (m) all combined, 12 and 18 months. Significant differences for

Macrod1 distance travelled only **b)** overall $F_{(1,107)}$=9.30, *p=0.003 and **d)** 18 month old female mice $F_{(1,27)}$=4.31, *p=0.047.

Table S9: Descriptive statistics of SQRT-fore and hind paw intensity for *Macrod1* from the catwalk gait analysis. SQRT = square root transformed. Mean ± SEM is presented. Individual N number is included as well as p-values for all terms and interactions. Statistics was performed on all factors combined using a factorial-ANOVA.

Macrod1 Catwalk		***SQRT-Forepaw Intensity (arbitrary)***		***SQRT-Hind Paw Intensity (arbitrary)***	
	AGE	WT	*Macrod1* KO	WT	*Macrod1* KO
Female	12 months	8.915 ± 0.117<br>N=13	8.8614 ± 0.0731<br>N=15	8.860 ± 0.137<br>N=13	8.7937 ± 0.0778<br>N=15
Male	12 months	9.3944 ± 0.0658<br>N=19	9.1560 ± 0.0924<br>N=14	9.0620 ± 0.0387<br>N=19	9.0842 ± 0.0861<br>N=14
Female	18 months	9.168 ± 0.133<br>N=16	8.9842 ± 0.087<br>N=13	8.879 ± 0.126<br>N=16	8.788 ± 0.109<br>N=13
Male	18 months	9.7048 ± 0.0676<br>N=12	9.4507 ± 0.0874<br>N=12	9.1770 ± 0.0709<br>N=12	9.090 ± 0.115<br>N=12
Genotype		$F_{(1,103)}$=0.03, p=0.859		$F_{(1,103)}$=0.64, p=0.426	
Sex		$F_{(1,103)}$=0.17, p=0.682		$F_{(1,103)}$=0.39, p=0.535	
Genotype*Sex		$F_{(1,103)}$=0.07, p=0.794		$F_{(1,103)}$=0.01, p=0.906	
Cohort		$F_{(1,103)}$=0.01, p=0.940		$F_{(1,103)}$=0.15, p=0.696	
Weight		$F_{(1,103)}$=13.15, *p < 0.001		$F_{(1,103)}$=0.02, p=0.902	
Weight*Sex		$F_{(1,103)}$=0.00, p=0.949		$F_{(1,103)}$=1.41, p=0.238	
Weight*Cohort		$F_{(1,103)}$=0.01, p=0.932		$F_{(1,103)}$=0.10, p=0.758	
Weight*Genotype		$F_{(1,103)}$=0.02, p=0.891		$F_{(1,103)}$=0.52, p=0.473	
Sex*Cohort		$F_{(1,103)}$=0.23, p=0.635		$F_{(1,103)}$=0.01, p=0.941	
Genotype*Cohort		$F_{(1,103)}$=0.11, p=0.739		$F_{(1,103)}$=0.65, p=0.424	

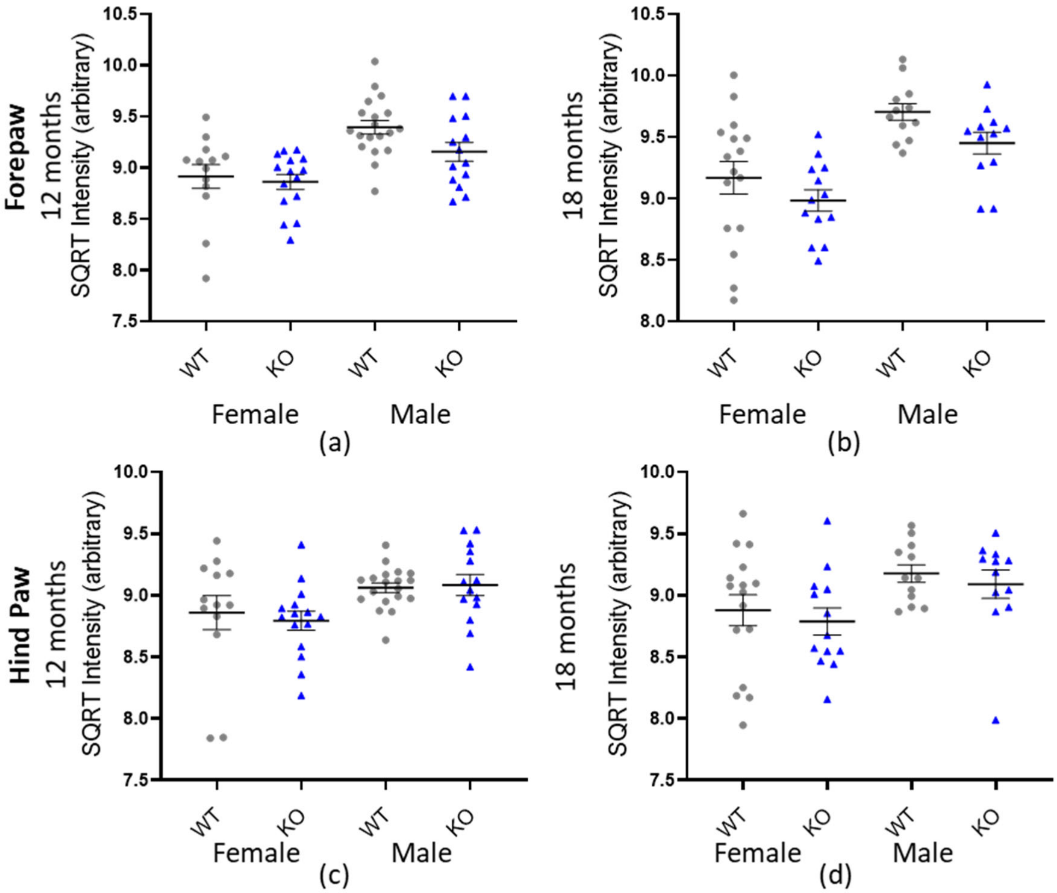

Figure S8: *Macrod1* KO is not associated with changes fore or hind paw foot pressure. Results from catwalk gait analysis. Graphs show individual values dot plot, WT shown in grey circles and Macrod1 KO in blue triangles. SQRT = Square-root transformed. Error bars are the SEM. Graphs are as follows, **a-b)** forepaw intensity at 12 and 18 months, **c-d)** hind paw intensity at 12 and 18 months.

Table S10: Descriptive statistics of gait velocity and SQRT gait cadence for *Macrod1* from the catwalk gait analysis. SQRT = square root transformed. Mean ± SEM is presented. Individual N number is included as well as p-values for all terms and interactions. Statistics was performed on all factors combined using a factorial-ANOVA.

***Macrod1* Catwalk**		***Gait Velocity (cm/s)***		***SQRT Gait Cadence (steps/minute)***	
	AGE	WT	*Macrod1* KO	WT	*Macrod1* KO
Female	12 months	23.13 ± 1.07<br>N=13	25.15 ± 1.92<br>N=15	3.9224 ± 0.0905<br>N=13	3.955 ± 0.102<br>N=15
Male	12 months	20.835 ± 0.709<br>N=19	21.25 ± 1.16<br>N=14	6.16 ± 0.0507<br>N=19	3.7590 ± 0.0592<br>N=14
Female	18 months	20.122 ± 0.800<br>N=16	21.75 ± 1.19<br>N=13	3.7547 ± 0.0613<br>N=16	3.7539 ± 0.0733<br>N=13
Male	18 months	20.716 ± 0.978<br>N=12	19.145 ± 0.919<br>N=12	3.6609 ± 0.0784<br>N=12	3.5930 ± 0.0594<br>N=12
Genotype		$F_{(1,103)}$=0.03, p=0.860		$F_{(1,103)}$=0.48, p=0.489	
Sex		$F_{(1,103)}$=0.09, p=0.767		$F_{(1,103)}$=0.00, p=0.991	

Genotype*Sex	$F_{(1,103)}$=2.04, p=0.156	$F_{(1,103)}$=2.28, p=0.134
Cohort	$F_{(1,103)}$=0.50, p=0.479	$F_{(1,103)}$=0.87, p=0.352
Weight	$F_{(1,103)}$=2.55, p=0.113	$F_{(1,103)}$=2.08, p=0.152
Weight*Sex	$F_{(1,103)}$=0.18, p=0.673	$F_{(1,103)}$=0.01, p=0.914
Weight*Cohort	$F_{(1,103)}$=0.26, p=0.613	$F_{(1,103)}$=0.32, p=0.573
Weight*Genotype	$F_{(1,103)}$=0.06, p=0.807	$F_{(1,103)}$=0.57, p=0.450
Sex*Cohort	$F_{(1,103)}$=0.49, p=0.483	$F_{(1,103)}$=0.30, p=0.583
Genotype*Cohort	$F_{(1,103)}$=0.49, p=0.486	$F_{(1,103)}$=0.03, p=0.859

Table S11: Descriptive statistics of forefoot swing speed and forefoot stride length for *Macrod1* from the catwalk gait analysis. Mean ± SEM is presented. Individual N number is included as well as p-values for all terms and interactions. Statistics was performed on all factors combined using a factorial-ANOVA.

***Macrod1* Catwalk**		***Forefoot Swing Speed (cm/s)***		***Forefoot Stride Length (cm)***	
	AGE	WT	*Macrod1* KO	WT	*Macrod1* KO
Female	12 months	54.34 ± 1.81 N=13	56.94 ± 3.08 N=15	5.995 ± 0.188 N=13	6.065 ± 0.191 N=15
Male		51.96 ± 1.03 N=19	52.26 ± 1.96 N=14	5.856 ± 0.126 N=19	5.813 ± 0.175 N=14
Female	18 months	50.92 ± 1.66 N=16	53.16 ± 2.26 N=13	6.134 ± 0.128 N=16	6.058 ± 0.138 N=13
Male		54.89 ± 1.87 N=12	49.08 ± 1.91 N=12	6.081 ± 0.170 N=12	5.736 ± 0.181 N=12
Genotype		$F_{(1,103)}$=0.35, p=0.558		$F_{(1,103)}$=0.01, p=0.939	
Sex		$F_{(1,103)}$=0.01, p=0.905		$F_{(1,103)}$=1.50, p=0.223	
Genotype*Sex		$F_{(1,103)}$=2.82, p=0.096		$F_{(1,103)}$=0.44, p=0.508	
Cohort		$F_{(1,103)}$=0.50, p=0.479		$F_{(1,103)}$=0.05, p=0.827	
Weight		$F_{(1,103)}$=0.00, p=0.970		$F_{(1,103)}$=0.52, p=0.473	
Weight*Sex		$F_{(1,103)}$=0.08, p=0.780		$F_{(1,103)}$=1.85, p=0.177	
Weight*Cohort		$F_{(1,103)}$=0.28, p=0.597		$F_{(1,103)}$=0.00, p=0.949	
Weight*Genotype		$F_{(1,103)}$=0.34, p=0.561		$F_{(1,103)}$=0.05, p=0.817	
Sex*Cohort		$F_{(1,103)}$=0.37, p=0.547		$F_{(1,103)}$=0.44, p=0.510	
Genotype*Cohort		$F_{(1,103)}$=1.40, p=0.240		$F_{(1,103)}$=0.51, p=0.476	

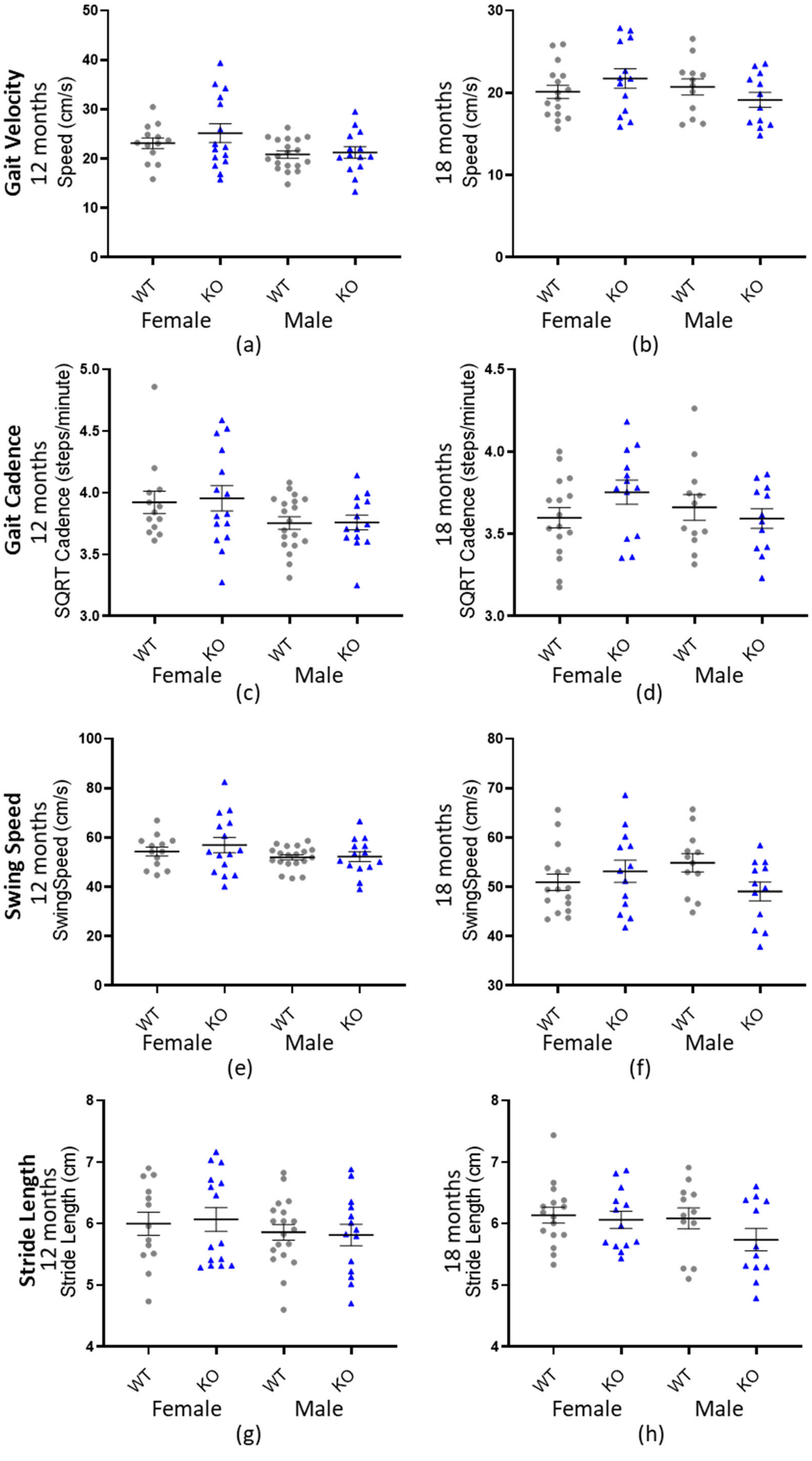

Gait Velocity
12 months
Speed (cm/s)
18 months
Speed (cm/s)
Gait Cadence
12 months
SQRT Cadence (steps/minute)
18 months
SQRT Cadence (steps/minute)
Swing Speed
12 months
SwingSpeed (cm/s)
18 months
SwingSpeed (cm/s)
Stride Length
12 months
Stride Length (cm)
18 months
Stride Length (cm)
WT
KO
WT
KO
Female
Male
(a)
(b)
(c)
(d)
(e)
(f)
(g)
(h)

Figure S9: *Macrod1* KO is not associated an altered gait. Results from catwalk gait analysis. Graphs show individual values dot plot, WT shown in grey circles and Macrod1 KO in blue triangles. SQRT = Square-root transformed. Error bars are the SEM. Graphs are as follows, **a-b)** gait velocity (average speed cm/s) at 12 and 18 months, **c-d)** SQRT gait cadence (steps/minute) at 12 and 18 months, **e-f)** Forefoot swing speed (cm/s) at 12 and 18 months, and **g-h)** forefoot stride length (cm) at 12 and 18 months.

Table S12: Descriptive statistics of SQRT-fore and hind paw intensity for *Macrod2* from the catwalk gait analysis. SQRT = square root transformed. Mean ± SEM is presented. Individual N number is included as well as p-values for all terms and interactions. *Macrod2* used a factorial-ANOVA with repeated measures, hence the term Mouse name. x = not an exact F-test.

Macrod2 Catwalk		***SQRT-Forepaw Intensity (arbitrary)***		***SQRT-Hind Paw Intensity (arbitrary)***	
	AGE	WT	*Macrod1* KO	WT	*Macrod1* KO
Female	3 months	8.6722 ± 0.0346<br>N=20	8.6733 ± 0.0522<br>N=17	8.9759 ± 0.0616<br>N=20	9.0381 ± 0.0578<br>N=17
Male		8.9180 ± 0.0705<br>N=13	8.8875 ± 0.0400<br>N=17	9.0652 ± 0.0811<br>N=13	9.1627 ± 0.0794<br>N=17
Female	13 months	8.1452 ± 0.0979<br>N=16	8.4966 ± 0.0942<br>N=16	7.959 ± 0.140<br>N=16	8.657 ± 0.116<br>N=16
Male		8.716 ± 0.104<br>N=13	8.302 ± 0.100<br>N=17	8.557 ± 0.137<br>N=13	8.230 ± 0.147<br>N=17
Genotype		$F_{(1,54)}$=0.46, p=0.501 x		$F_{(1,54)}$=1.54, p=0.219 x	
Sex		$F_{(1,54)}$=0.56, p=0.456 x		$F_{(1,54)}$=0.56, p=0.456 x	
Genotype*Sex		$F_{(1,54)}$=8.31, *p=0.005 x		$F_{(1,54)}$=7.47, p=0.008 x	
Cohort		$F_{(1,54)}$=1.99, p=0.164		$F_{(1,54)}$=3.79, p=0.057	
Weight		$F_{(1,54)}$=1.19, p=0.280		$F_{(1,54)}$=0.01, p=0.935	
Weight*Sex		$F_{(1,54)}$=0.51, p=0.479		$F_{(1,54)}$=0.04, p=0.835	
Weight*Cohort		$F_{(1,54)}$=0.20, p=0.657		$F_{(1,54)}$=1.06, p=0.309	
Sex*Cohort		$F_{(1,54)}$=1.21, p=0.276		$F_{(1,54)}$=0.81, p=0.373	
Genotype*Cohort		$F_{(1,54)}$=0.01, p=0.936		$F_{(1,54)}$=1.80, p=0.186	
Mouse Name		$F_{(1,54)}$=0.96, p=0.558		$F_{(1,54)}$=1.30, p=0.158	

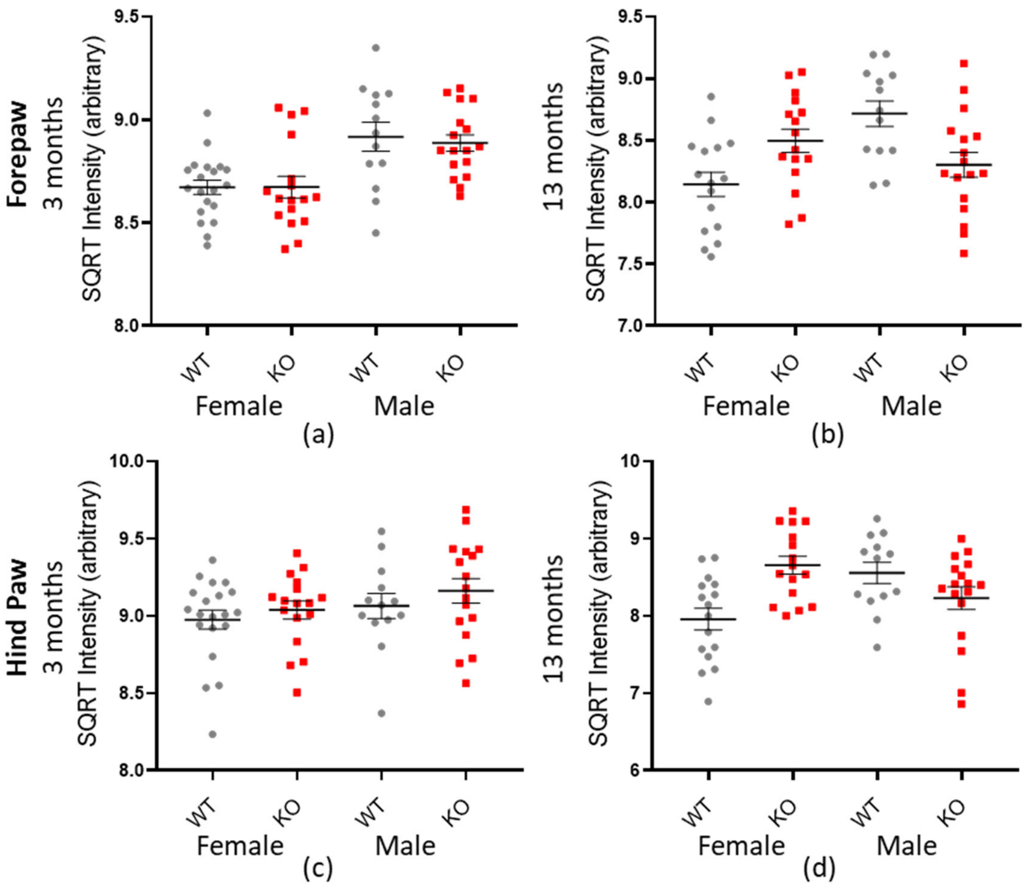

Figure S10: *Macrod2* KO is not associated with changes in fore or hind paw foot pressure. Results from catwalk gait analysis. Graphs show individual values dot plot, WT shown in grey circles and Macrod2 KO in red squares. SQRT = Square root transformed. Error bars are the SEM. Graphs are as follows, **a-b)** forepaw intensity at 3 and 13 months, **c-d)** hind paw intensity at 3 and 13 months.

Table S13: Descriptive statistics of *Macrod1* grip strength. Results from 4 limb hang test. SQRT = square root transformed. Mean ± SEM is presented. Individual N number is included as well as p-values for all terms and interactions. Statistics was performed on all factors combined using a factorial-ANOVA.

***Macrod1* Grip Strength**	***SQRT Hanging Impulse (gs)***		
	AGE	WT	*Macrod1* KO
Female	12 months	76.94 ± 6.68 N=13	79.72 ± 4.54 N=17
Male	12 months	60.14 ± 3.80 N=19	63.85 ± 5.03 N=15
Female	18 months	40.27 ± 1.75 N=17	45.98 ± 4.25 N=15
Male	18 months	35.13 ± 3.21 N=12	43.06 ± 3.27 N=12
Genotype		$F_{(1,109)}$=0.51, p=0.477	
Sex		$F_{(1,109)}$=1.05, p=0.307	
Genotype*Sex		$F_{(1,109)}$=0.40, p=0.529	
Cohort		$F_{(1,109)}$=19.89, *p > 0.001	
Weight		$F_{(1,109)}$=25.43, *p > 0.001	
Weight*Sex		$F_{(1,109)}$=1.44, p=0.234	
Weight*Cohort		$F_{(1,109)}$=11.92, *p=0.001	
Weight*Genotype		$F_{(1,109)}$=0.76, p=0.384	
Sex*Cohort		$F_{(1,109)}$=1.28, p=0.261	
Genotype*Cohort		$F_{(1,109)}$=0.19, p=0.662	

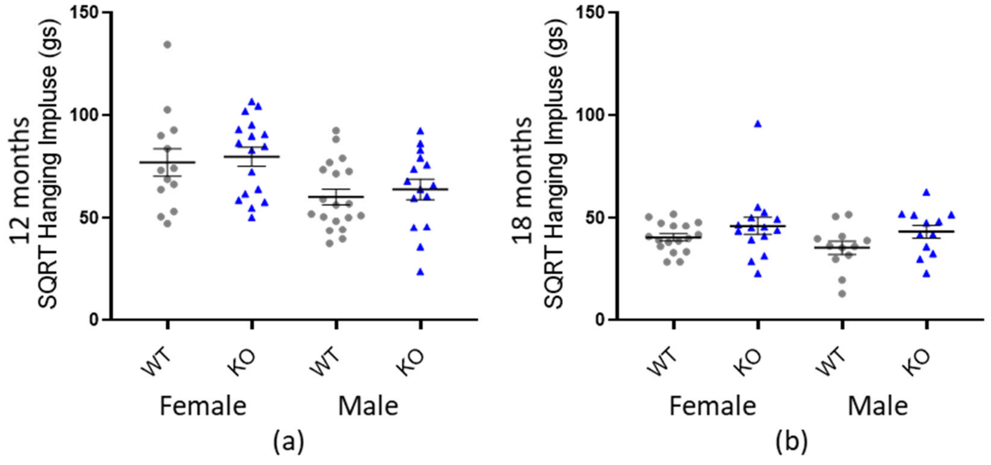

Figure S11: *Macrod1* KO is not associated altered grip strength. Results from 4 limb hang test. Graphs show individual values dot plot, WT shown in grey circles and Macrod1 KO in blue triangles. SQRT = Square root transformed. Error bars are the SEM. Graphs are as follows, **a-b)** SQRT hanging impulse (gs) at 12 and 18 months.

Table 14: Descriptive statistics of *Macrod2* grip strength. Results from link lifting test. Mean ± SEM is presented. Individual N number is included as well as p-values for all terms and interactions. *Macrod2* used a factorial-ANOVA with repeated measures, hence the term Mouse name. x = not an exact F-test.

***Macrod2* Grip Strength**	***Lift Score (out of 20)***		
	AGE	WT	*Macrod2* KO
Female	3 months	9.606 ± 0.963 N=20	11.149 ± 0.927 N=17
Male	3 months	7.995 ± 0.818 N=13	8.082 ± 0.578 N=16
Female	13 months	5.407 ± 0.820 N=16	6.45 ± 1.01 N=16
Male	13 months	5.464 ± 0.404 N=13	4.900 ± 0.289 N=17
Genotype		$F_{(1,53)}$=0.03, p=0.866 x	
Sex		$F_{(1,53)}$=0.13, p=0.721 x	
Genotype*Sex		$F_{(1,53)}$=1.69, p=0.198 x	
Cohort		$F_{(1,53)}$=3.26, p=0.077	
Weight		$F_{(1,53)}$=1.83, p=0.182	
Weight*Sex		$F_{(1,53)}$=0.04, p=0.846	
Weight*Cohort		$F_{(1,53)}$=1.94, p=0.170	
Sex*Cohort		$F_{(1,53)}$=0.19, p=0.667	
Genotype*Cohort		$F_{(1,53)}$=0.50, p=0.483	
Mouse name		$F_{(1,53)}$=1.55, p=0.052	

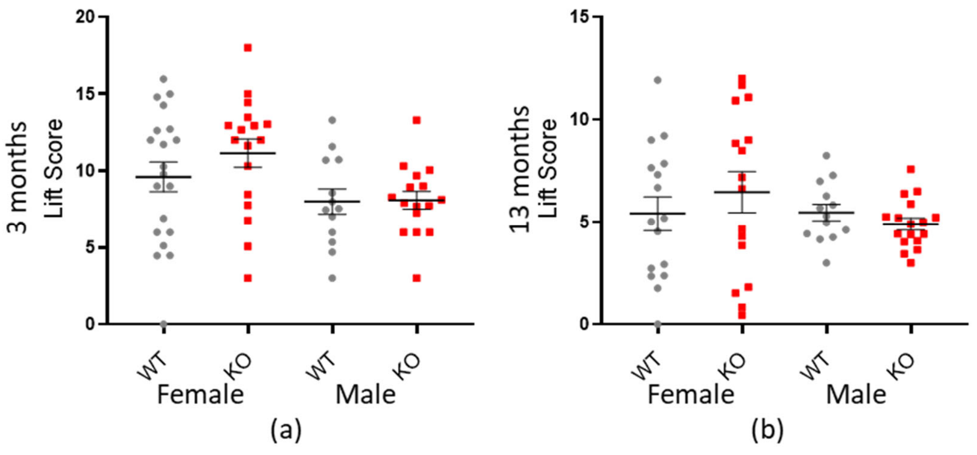

Figure S12: *Macrod2* KO is not associated altered grip strength. Results from link lifting test. Graphs show individual values dot plot, WT shown in grey circles and Macrod2 KO in red squares. Error bars are the SEM. Graphs are as follows, **a-b)** Lift score at 3 and 13 months.
